# Bio-Microcapsules of Polybutylene Succinate (PBS) and Isocyanates: Towards Sustainable, Safer, and Efficient Adhesives

**DOI:** 10.3390/polym17020139

**Published:** 2025-01-08

**Authors:** Lucas P. Marcelino, António Aguiar, Rui Galhano dos Santos, Isabel Pinho, Ana C. Marques

**Affiliations:** 1 CERENA-Centro de Recursos Naturais e Ambiente, Department of Chemical Engineering (DEQ), Instituto Superior Técnico, Universidade de Lisboa, Avenida Rovisco Pais, 1049-001 Lisboa, Portugal; lucas.marcelino@tecnico.ulisboa.pt (L.P.M.); rui.galhano@tecnico.ulisboa.pt (R.G.d.S.); 2 CIPADE S.A., Av. Primeiro de Maio 121, 3700-227 São João da Madeira, Portugal

**Keywords:** microcapsules, isocyanate, bio-PBS, adhesives

## Abstract

This work describes the encapsulation of three different aliphatic isocyanates to reduce the risks associated with isocyanates’ direct handling. The use of bio-based polybutylene succinate (bio-PBS) increases the sustainability factor as it allows for the use of microcapsules (MCs) from renewable sources with biodegradable features. The three different MCs (MCs-Monomer, MCs-Trimer, and MCs-Polymer) are spherical, crack-free, and matrix-type, containing an isocyanate payload between 67 wt% and 70 wt%. Protection against environmental moisture was improved, resulting in losses of less than 10% for most cases after one month. The bio-PBS MCs were found to be suitable as crosslinking agents in high-performance adhesive formulations for the footwear industry. Adhesive joints with encapsulated isocyanate exhibited peel strength values ranging from 3.28 to 4.56 N/mm, well above the minimum requirements for the intended footwear application. Additionally, these joints demonstrated improved creep resistance compared to those using non-encapsulated isocyanates. In this context, the MCs-Trimer stood out, providing exceptional thermal robustness to the joints, as they showed no failure or opening at 90 °C, consistent with commercial adhesives. These results confirm that bio-PBS MCs can be excellent components for future adhesive formulations and that matrix-type MCs can also be utilised for this purpose.

## 1. Introduction

Isocyanates are a family of chemical compounds characterised by the -NCO group and were first synthesised in 1848 by Wurtz [1]. They are highly reactive towards common nucleophiles like -OH, -NH, and -SH, and are primarily used in the production of polyurethane for foams, coatings, and adhesives [1,2,3]. In the footwear industry, isocyanates are used as a monomer for polyurethane soles or upper [4,5] and as a crosslinking agent in adhesive formulations [6]. Despite their common use and advantages, isocyanates are toxic compounds. Prolonged exposure to isocyanate vapours and/or aerosols in the workplace environment can lead to various symptoms in workers since they are powerful irritants of the mucous of the eyes and respiratory tract [7]. The more common consequence is the development of respiratory illnesses such as occupational asthma, bronchitis, and rhinitis [8,9]. The European Commission has been actively engaged in limiting the direct use of isocyanates to safeguard workers, and, in 2023, regulations were implemented to ensure the safe handling of this compound [10].

Microencapsulation is the process of enclosing a solid, liquid, or gas to produce small particles called microcapsules (MCs) with a size between a few microns and a few millimetres [11]. This technology is used for different applications, such as food technology [11,12,13,14], fragrances [15,16,17,18], the pharmaceutical industry [11,19,20,21], agriculture [22,23,24], and energy storage [25,26,27]. The microencapsulation of isocyanate species has emerged as a valuable solution with dual benefits since it offers a protective shield around isocyanate molecules and ensures the safety of workers and end-users by preventing direct contact. By encapsulating isocyanates, the risk of premature reactions or degradation is also minimised, increasing the shelf life and reliability of these chemical compounds. Therefore, the microencapsulation of isocyanates is an approach with great potential and has the benefit of complying with the recent regulation on isocyanates [10].

Isocyanate encapsulation for adhesives started being published more specifically for epoxy resins and using polymers such as polymethacrylates as the encapsulant for self-healing metal joints [28,29]. Nowadays, the vast majority of the literature on the microencapsulation of isocyanates focuses on developing materials for self-healing and anti-corrosion purposes [30,31,32,33], and very few studies focus on adhesives [34,35]. In the context of footwear applications, some approaches have been explored, but interfacial polymerisation is the most used production technique, and isophorone diisocyanate (IPDI) is the most studied isocyanate monomer [36,37].

Due to the rigidity of the polyurethane and/or polyurea shell, obtained by interfacial polymerisation, polyurethane, and/or polyurea, MCs are more difficult to break and hamper the release of the isocyanate content. Additionally, these polymers require more isocyanate, typically produced from fossil-based raw materials, and have very limited biodegradability. Therefore, alternative polymers have begun to be studied with a focus on applicability and sustainability. MCs of polycaprolactone (PCL) with IPDI were the first example of biodegradable MCs containing isocyanates, followed by two other types of MCs: polycaprolactone/polylactic acid (PCL/PLA) and polybutylene adipate terephthalate (PBAT) loaded with IPDI [38,39,40].

PBS is a polyester produced using succinic acid and 1,4-butanediol. These monomers are still commonly fossil-based but can also be obtained from natural resources such as corn and sugarcane, making it bio-based [41,42]. PBS is a white semi-crystalline thermoplastic with a 1.25 g/cm^3^ density. It exhibits a melting point within the 90–120 °C range and a low glass transition temperature, typically around −45 °C to −10 °C. The Young’s Modulus varies among 300–500 MPa, depending on the degree of crystallinity. PBS has been applied in different fields such as agriculture, packaging, and biomedicine due to its good film-forming ability [41,43]. Its biodegradability is especially attractive for single-use plastics because it degrades at a high rate during a short period of time [44,45,46]. When designing MCs for this application, in what concerns selecting the shell’s material, PBS stands out from the other polyesters because it is not as brittle as PLA or polyhydroxybutyrate (PHB), but it exhibits less elongation at break than PCL and PBAT, as it is able to break during the application and releasing of the core content as desired. Despite its potential, the use of PBS as an encapsulating material is still quite limited, with just a few published works focusing on drug delivery [47,48,49,50] and agriculture [51,52].

In this study, the solvent evaporation technique was used to produce bio-based microcapsules (bio-MCs) by using bio-PBS to encapsulate a series of aliphatic isocyanates derived from hexamethylene diisocyanate (HDI), which is more reactive than IPDI and, therefore, more adequate for high-performance adhesives. The main objectives of this work were to improve the manufacturing process of bio-based and biodegradable MCs, enhance isocyanate encapsulation, and thoroughly characterise the morphology and loading of the final MCs. Furthermore, the MCs were integrated into adhesive formulations, and the resulting adhesive joints were tested to evaluate their mechanical and thermal resistance, which were compared with their non-encapsulated isocyanate counterparts.

## 2. Materials and Methods

### 2.1. Materials

The isocyanates, namely the monomeric HDI (Desmodur^®^ H), the trimer (Desmodur^®^ N3900), and the polymer (Desmodur^®^ DN), were obtained from Covestro AG (Leverkusen, Germany). Chloroform (>99.8%, HPLC grade) was supplied by Fisher Chemical (Porto Salvo, Portugal), and bio-PBS FZ91PM was provided by PPT MCC Biochem Company Limited (Bangkok, Thailand). The polyvinyl alcohol (PVA) (Selvol 540, 87–88% hydrolysed) was supplied by Sekisui Speciality Chemical Company (Dallas, TX, USA). The polyurethane-based adhesive, commercially known as Plastik 6275, was provided by CIPADE S.A. (São João da Madeira, Portugal). All products were used without further purification.

### 2.2. Production of Bio-Microcapsules (Bio-MCs) of PBS Loaded with Isocyanates

The MCs were produced by a combination of an oil-in-water emulsion (O/W) and solvent evaporation technique using bio-PBS. The PBS used is bio-based and biodegradable, so the MCs discussed in this work are classified as bio-MCs.

**Figure 1:**
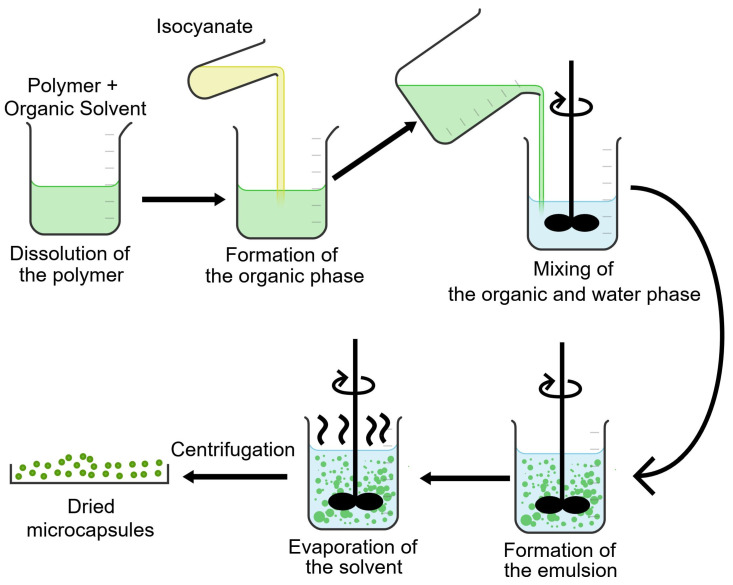
Production of PBS bio-MCs loaded with isocyanates.

As a standard production protocol, we consider the following procedure: at room temperature, 2.25 g of PBS was dissolved in 25 g of chloroform and magnetically stirred until all pellets were dissolved. Then, the PBS solution was added to 4.7 g of the isocyanate species and mixed for 5 min, forming the organic phase (OP). Simultaneously, for the water phase (WP), a 2 wt% solution of PVA with a total weight of 50 g was prepared. Subsequently, the OP was added to the WP while subject to mechanical stirring (750 rpm) with the aid of a helix-shaped stirring impeller (J.P. Selecta, Abrera, Barcelona, Spain), as illustrated in Figure 1. The emulsion was left stirring for 4 h and the MCs were vacuum-filtrated. The collected MCs were then thoroughly washed with deionised water multiple times, air-dried for 24 h, and stored at room temperature with a 40–60% humidity range.

A parameter variation study was conducted to investigate the effects of parameters on the morphology and load of the MCs. The influence of the PVA in the WP was tested by using three different concentrations of PVA (1, 2, and 3 wt%) and the influence of the amount of isocyanate capable of being encapsulated by the polymer was tested by changing the amount of isocyanate present in the OP (8, 15 and 20 wt%). The other production parameters remained unchanged.

### 2.3. Characterisation of the Microcapsules

The morphology of the MCs was monitored using an optical microscope (KERN OZP 558, Kern & Sohn, Balingen, Germany). The surface of the MCs was also examined with a scanning electron microscope (Phenom ProX G6, Thermo Fisher Scientific, Waltham, MA, USA) to evaluate the presence of cracks or holes and determine their typology. Additionally, microscopy played an important role in determining the diameter of the MCs. The diameters were measured using the Fiji software (ImageJ/Fiji 1.53k) using data sets of at least 250 measurements and manual tracing for precise measurements. The diameters are represented by their mean average values, and by D5, median (D50), and D95, which are presented in box plots. Statistical analysis was performed and determined by nonparametric analysis (Kruskal–Wallis test and Dwass–Steel–Critchlow–Fligner test) using Jamovi software, version 2.3.28. A statistically significant difference between data is considered when *p* < 0.05.

A Spectrum Two FT-IR spectrometer from PerkinElmer (Waltham, MA, USA) was used to obtain FTIR spectra and was equipped with a UATR Two accessory. The spectra were captured with a resolution of 4 cm^−1^ and data accumulation of 16 scans.

Thermogravimetric analysis (TGA, Hitachi STA 7200 thermal analysis system (Ibaraki, Japan)) was performed using 5 to 10 mg of sample which was put into an aluminium pan and heated from 35 to 600 °C at a rate of 10 °C/min with a nitrogen feed rate of 200 mL/min.

The TGA-FTIR analysis was performed using simultaneous TGA (TGA STA8000, PerkinElmer) coupled with FTIR spectrometer (PerkinElmer). Approximately 15 mg of the sample was heated under a nitrogen gas flow rate of 200 mL/min from 35 °C to 600 °C at a heating rate of 10 °C/min. The gases were transported to the FTIR spectrometer through a connecting line heated at 275 °C to prevent the evolved gases from condensing. The FTIR spectra were recorded at a resolution of 8 cm^−1^ from 4000 to 500 cm^−1^, and 8 scans were conducted for each spectrum.

DSC (Differential Scanning Calorimetry) tests were carried out using the DSC 4000 (PerkinElmer, Waltham, MA, USA). The samples were put into an aluminium pan and heated from 0 to 150 °C at a rate of 10 °C/min with a nitrogen feed rate of 20 mL/min.

Powder X-ray Diffraction (XRD) was performed using the Bruker D2 Phaser (Karlsruhe, Germany). The diffractograms were obtained within an interval of 10 to 40° 2θ degrees with an increment of 0.03° using a K-a (Cu) radiation lamp with a wavelength of 1.5406 Å.

^1^H NMR spectra were acquired in a Bruker Avance III 400 spectrometer (Karlsruhe, Germany) with deuterated chloroform (CDCl_3_) as solvent. The samples were prepared immediately prior to their assessment and an internal standard (4-chloro-3-methylphenol) was used. The signals at 6.50–6.75 ppm from the internal standard and the isocyanates’ signals at 3.3 ppm (HDI) or 3.8 ppm (trimer or polymer) were selected for direct correlation [53]. Equation (1) was used to quantify the isocyanate loading (Iso wt%) except for the isocyanate polymer. “n_std_” stands for the mol value of the standard; “k” is a theoretical ratio between the signal of the used standard and isocyanate when the amount of both is equivalent (k = 0.5 for HDI and 0.4 for HDI based-trimer); “M_isocyanate_” stands for the molecular weight of the isocyanate; “r” is the ratio obtained between the signals; and “_mMCs_” refers to the mass of the MCs. The quantification of the isocyanate polymer was performed using a calibration curve (Figures S1 and S2).

(1) $$Iso\left(wt\%\right)=\frac{n_{std}\times k\times M_{isocyanate}}{r\times m_{MCs}}\times 100$$

### 2.4. Behaviour of MCs over Time and Dispersed in Solvents

The MCs were kept in an environment with relative humidity between 40% and 50% at room temperature (18–25 °C) and the isocyanate content was measured using TGA and NMR after 1, 2, and 3 months. The behaviour of the MCs in different solvents was studied to analyse their effect on the MCs in terms of isocyanate content evolution along the time of exposure to the solvent. For this, 0.1 g of MCs was added in 5 mL of different solvents (water, ethyl acetate, acetone, and hexane) and characterised after 1 day, 3 days, and 1 week, adapting the protocol from [40].

### 2.5. Production and Characterisation of the Adhesive Joints

For adhesive production, the crosslinker (isocyanate), at 5 wt%, was mixed with Plastik 6275 adhesive. This percentage was selected based on earlier findings that demonstrated superior results compared to concentrations of 2.5 wt% or 7.5 wt% of other isocyanates [39]. Pairs of substrates with dimensions of 13 cm × 3 cm of Neolite, a synthetic rubber commonly used to replace leather, were subjected to a chemical abrasion treatment with 2190 Halinov (CIPADE S.A.). The adhesive formulation was applied on the substrates with a brush in an area around 10 cm × 3 cm and allowed to dry for 15 min at room temperature. The films formed on the substrates were activated by IR radiation for 6 s at around 70 °C. Then, the substrates were bonded and subjected to a pressure of 4 bar for 10 s. Afterwards, the adhesive joints were stored for a week in standard conditions (23 °C, 50% relative humidity) to guarantee that the cure of the adhesive was complete. The peel strength test was executed using a universal testing machine Instron 5566 and following the standard EN 1392 [45]. Each test was repeated three times at an angle of 180° and a crosshead speed of 100 mm/min. The creep test was performed while the specimen was suspended at different temperatures for 2 h with a weight of 300 g, starting at 60 °C and increasing in steps of 10 °C until reaching 90 °C. The displacement was measured every two hours. For every test, three adhesive joints (replicates) were considered.

## 3. Results and Discussion

### 3.1. Study of the Impact of the Production Parameters on Bio-PBS MC Production

The limited research on microencapsulation using PBS underscores the importance of studying this system and optimising its production parameters. The major requirements critical for high-quality MCs and their employment in adhesives are related to the MCs’ size, stability, and isocyanate loading. Based on previous studies [39,54], the MCs for the intended adhesives should ideally have a 50–80 µm diameter to ensure ease of production, good dispersibility within the adhesive joint, and, mainly, to facilitate the targeted breakage with the release of isocyanate during the adhesive joint preparation. They should exhibit minimal agglomeration and maintain a loose structure, ensuring they disperse easily when applied to the substrate. While smaller MCs might theoretically offer a more uniform distribution, they are prone to forming more aggregates, which negatively impacts the release and protection of the isocyanate [40]. Additionally, the MCs should contain a high isocyanate content, above 50 wt%, to minimise the polymer (shell) content in the joint, which could otherwise act as a defect and hinder the crosslinking between the adhesive and isocyanate [40]. Regarding chemical stability, the formation of polyurea during both production and storage is undesirable because it indicates that some of the isocyanate content is reacting prematurely. HDI was the isocyanate selected in this study due to its widespread use, simplicity, high reactivity, and role as the base compound for trimer and polymeric isocyanates [54].

#### 3.1.1. Changing the Amount of PVA

Emulsifiers are used to reduce the interfacial tension between the continuous and dispersed phases. Typically, as the emulsifier concentration increases, the droplet size decreases. However, increasing the emulsifier concentration beyond a certain point has little to no further impact on the interfacial tension. At this point, the risk of depletion flocculation increases, which may promote particle coalescence [55].

Several studies have reported PVA as an emulsifier [56,57,58], but PVA works mainly as an emulsion stabiliser. As an emulsifier, PVA reduces the interfacial tension between the oil and water phases, aiding in the formation of smaller droplets. As an emulsion stabiliser, PVA enhances the long-term stability of the emulsion by preventing droplet aggregation and phase separation. This stabilising effect is primarily due to steric stabilisation, where PVA molecules form a physical barrier around the droplets, preventing them from coalescing. Furthermore, PVA increases the viscosity of the aqueous phase, slowing down the movement of the droplets and reducing the tendency for them to aggregate.

**Figure 2:**
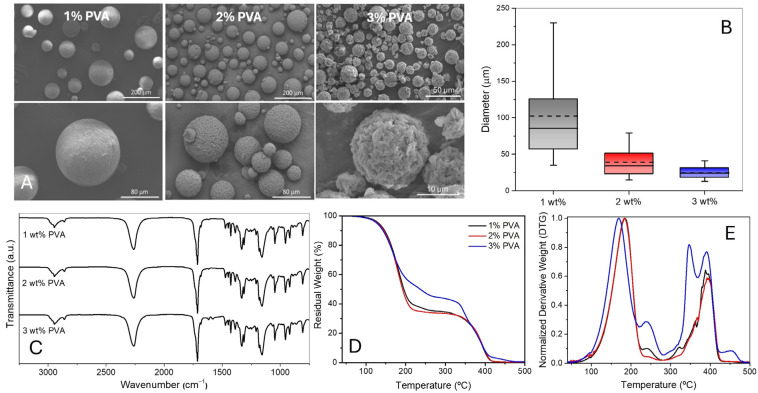
(**A**) SEM images of the MCs produced using different PVA concentrations; (**B**) box charts with the median and average diameter of the MCs; (**C**) FTIR spectra of the MCs; (**D**) TGA (residual weight versus temperature); and (**E**) DTG of the MCs, percentage of isocyanate equal to 63 wt%, 67 wt%, and 52 wt% for 1 wt%, 2 wt%, and 3 wt% PVA, respectively.

This study tested three different concentrations of the PVA (1, 2, and 3 wt% of WP), with the amounts of the other components remaining unchanged (4.7 g of HDI, 2.25 g of bio-PBS, 25 g of chloroform, 50 g of WP). As shown in Figure 2A and Figure S3, the MCs become smaller as the PVA content in the water phase increases. The impact of varying the PVA concentration on the final diameter of MCs is substantial. Specifically, increasing the PVA from 1 wt% to 2 wt% results in a reduction of approximately 60% in both the mean and median diameters, while a further increase from 2 wt% to 3 wt% leads to a decrease of around 30% (Figure 2B). Furthermore, their surface also becomes rougher as their size decreases. As shown in Table S1, the differences in the sizes of the MCs are statistically significant.

Through the FTIR spectra (Figure 2C), the encapsulation of isocyanate is evident. The MCs’ spectra exhibit the characteristic bands of both bio-PBS and HDI (Figures S4 and S5). The band at approximately 2260 cm^−1^, ascribed to N=C=O stretching, confirms the presence of HDI. The peak at 1354 cm^−1^ is related to the asymmetric bending vibration of CH_2_ attached to an NCO group. The specific peak of PBS at 1710 cm^−1^ is attributed to the stretching of C=O of the ester group. The stretching of the -C-O-C- link is observed at 1140 cm^−1^, and the peak at 1045 cm^−1^ corresponds to the stretching of the -O-C-C bond. The vibration of -CH_2_ groups is assigned to the peak at 1330 cm^−1^. The spectra of the MCs obtained for higher percentages of PVA slightly show the presence of polyurea (PUa), revealed by the peaks at ca. 1617 cm^−1^ and 1580 cm^−1^, which correspond to the stretching of the C=O and N–H groups, respectively (Figure S6).

TGA (Figure 2D) can provide more precise information about the percentage of encapsulated isocyanate and polyurea (PUa) formation. By analysing the thermograms, we conclude that, in general, they represent a blend of the raw materials (Figure S7) and that the percentage of HDI ranges between 67 wt% and 52 wt% (T_max_ at 180 °C). The thermograms also reveal that a higher amount of PVA leads to a smaller amount of encapsulated isocyanate (55%) and increased formation of PUa (T_max_ at 355 and 390 °C). These new thermal events, which are much more evident in the MCs formed with 3 wt% PVA, correspond to the PUa formed by the reaction of HDI and H_2_O. The MCs obtained with 1 wt% PVA show only a slight loss of HDI, with an HDI loading of about 65 wt%. The MCs produced with 2 wt% PVA show a slightly better preservation of HDI, with a loading of 67 wt%, which matches the theoretical maximum loading of 67 wt%, calculated by dividing the mass of HDI used by the total mass of HDI and bio-PBS. This suggests that the reaction between water and isocyanate is facilitated as the droplet size decreases and its surface area increases. So, with the decrease in size, the content of PUa is expected to increase. The MCs obtained with 1 and 2 wt% also show a slight improvement in thermal stability, as the onset and T_max_ related to isocyanate loss are shifted 12 °C to higher temperatures. Based on the size distribution, morphology, and analysis of the spectra and thermograms, the MCs produced with 2 wt% PVA are the most suitable in terms of MCs’ size, stability, and isocyanate loading.

#### 3.1.2. Changing the Amount of Isocyanate

**Figure 3:**
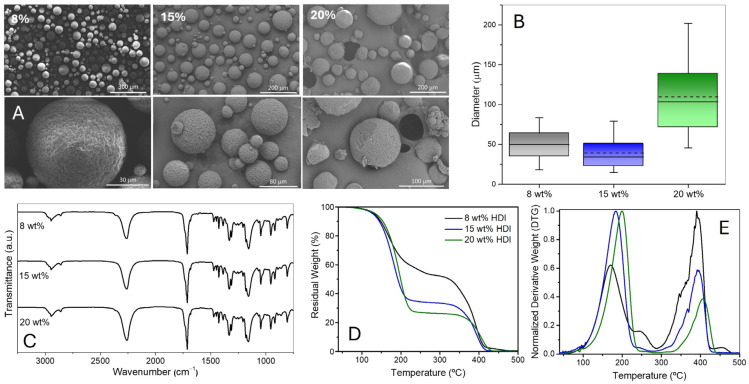
(**A**) SEM images of the MCs produced using different concentrations of isocyanate; (**B**) box charts with the median and average diameter of the MCs; (**C**) FTIR spectra of the MCs; (**D**) TGA (residual weight versus temperature); and (**E**) DTG of the MCs, percentage of isocyanate equal to 47 wt%, 67 wt%, and 73 wt% for 8 wt%, 15 wt%, and 20 wt% HDI, respectively.

The maximum amount of isocyanate that can be encapsulated is crucial for improving the final product. In this study, we assessed three different quantities of HDI, ranging from 8% to 20% in the OP (between 2.7 g and 6.7 g), while the other components remained unchanged (2 wt% of PVA in 50 g of WP, 2.25 g of bio-PBS, and 25 g of chloroform). The resulting MCs (Figure 3A) exhibit a spherical shape without holes or cracks. The impact of increasing HDI content on droplet size (Figure 3B) is not straightforward due to two opposing factors. HDI, with its relatively low viscosity (3 mPa·s), reduces the viscosity of the OP (dispersed phase), promoting a more homogeneous mixture of the droplets, less droplet coalescence, and, consequently, smaller MCs (Figure S8). This is stated by the differences between 8 wt% and 15 wt% of HDI. However, beyond a certain concentration, the increased isocyanate content disrupts the balance between polymer–isocyanate–solvent interactions, causing some destabilisation of the emulsion and resulting in larger MCs. Additionally, as the amount of encapsulated HDI rises, a ballooning effect can occur, which, when pushed to extremes, may lead to droplet bursting. These factors likely contribute to a significant increase in MCs’ size when the content of HDI increases from 15 to 20 wt%. As shown in Table S2, the differences in the sizes of the MCs are statistically significant.

As previously observed, the FTIR spectra (Figure 3C) show the typical bands of isocyanate and PBS. In this case, the MCs obtained with 8 wt% HDI display low-intensity bands associated with the C=O and N–H groups of PUa. The TGA (Figure 3D) confirms that there is around 8 wt% PUa content in the MCs produced with the lowest amount of HDI. The obtained values for the loading of HDI show that both 15 and 20 wt% have loadings near the theoretical loading. Considering these results, we conclude that the combination of 2 wt% PVA and 15 wt% HDI enables the production of small and loose MCs with high HDI loading (67 wt%), while 20 wt% HDI allows an isocyanate loading as high as ca. 73%; but, the resulting average particles’ size above 100 µm presents a drawback.

For core-shell MCs (single core of the active compound wrapped in a protective shell) formed through solvent evaporation, during the initial stages of emulsion formation, the solvent present at the droplet interface diffuses into the continuous phase, leaving a polymer-rich layer at the interface. As more solvent diffuses and evaporates, the system crosses the binodal line, entering a metastable phase where the nucleation and growth of polymer-rich regions occur within the droplet and, more specifically, in the water–droplet interface, where its concentration is already high [59]. Due to the formation of a membrane at the interface, small polymer domains within the droplet migrate toward the surface, where polymer nucleation has already started. This process facilitates the heterogeneous nucleation and growth of the polymer-rich phase at the droplet’s interface, pushing the species to be encapsulated towards the core, where the polymer content is lower.

**Figure 4:**
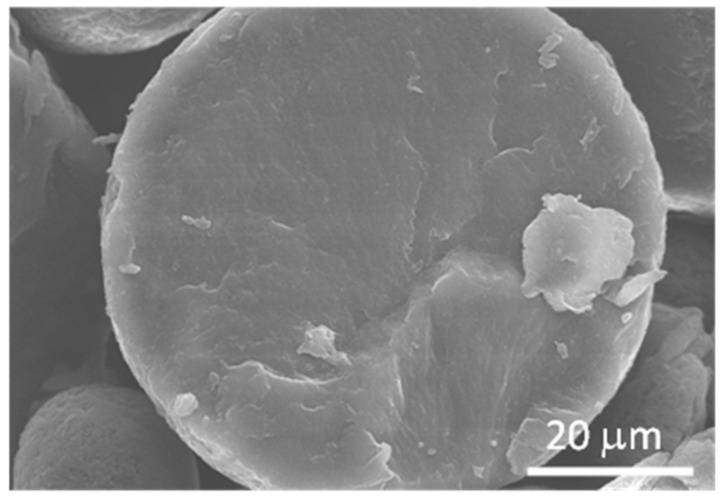
Cross-section of the PBS MCs demonstrating the matrix-type structure of the MCs.

In matrix-type MCs, the active compound is homogeneously distributed throughout the matrix material without a distinct boundary. The formation of matrix-type MCs follows a different process, as spinodal decomposition causes the polymer and active compound to separate spontaneously, preventing the development of well-defined regions. This occurs due to weak interactions between the polymer and the other components. SEM analysis of the bio-PBS MCs loaded with HDI shows that the MCs exhibit a matrix-type structure. This finding was consistent across multiple examples, including those containing other aliphatic isocyanates, discussed later in this work. As depicted in Figure 4, the cross-sectional view of an MC demonstrates a uniform distribution of both the polymer and isocyanate throughout the particle without noticeable segregation into polymer-rich or isocyanate-rich domains. Matrix-type MCs tend to be more mechanically robust than core-shell structures, and, to our knowledge, no previous studies have shown that isocyanate is encapsulated in matrix-type MCs.

### 3.2. Production of Microcapsules with Different Aliphatic Isocyanates (HDI-Monomer, HDI-Trimer, and HDI-Polymer)

As previously mentioned, the better conditions for synthesising bio-PBS MCs with HDI involved a stirring speed of 750 rpm, 50 g of WP, 2.25 g of bio-PBS, 25 g of CHCl_3_, 2 wt% (1 g) of PVA, and 15 wt% (4.7 g) of HDI. These parameters were used to encapsulate both the HDI-trimer and HDI-polymer. However, it is important to note that the best conditions for encapsulating HDI may not be the best for other HDI derivatives. Regarding the trimer, it should also be highlighted that, instead of the typical symmetric HDI-trimer (isocyanurate ring), in this work, we used the asymmetric trimer (imminooxadiazinedione ring), which retains the same functionality as the symmetric trimer but is four times less viscous. This isocyanate is not only relatively recent and still underexplored, but it also exhibits greater reactivity than its isomer with the isocyanurate ring [60], making it a promising candidate for use as a crosslinker in adhesives. To the best of our knowledge, it has never been encapsulated or employed in adhesive formulations.

**Figure 5:**
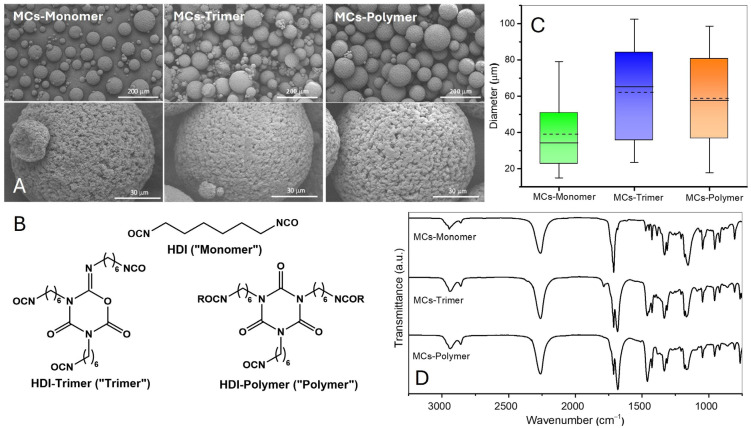
(**A**) SEM images of the MCs produced using different aliphatic isocyanates; (**B**) chemical structures of the encapsulated isocyanates; (**C**) box charts with the median and average diameter of the MCs; (**D**) FTIR spectra of the MCs.

Figure 5A shows the successful formation of MCs with the three different isocyanates. The MCs are all spherical, loose, and have a rough surface. For the trimer, the viscosity is around 730 mPa·s, and for the polymer, about 1250 mPa·s, values well above to that of the monomer, 3 mPa·s. The increase in viscosity favours the formation of bigger droplets and, consequently, the size of the final MCs (Figure 5C). There is no significant difference between MCs-Trimer and MCs-Polymer, since the differences are not statistically significant (Table S3 and Figure S9).

The chemical characterisation of these MCs was performed by ^1^H NMR, FTIR, and TGA. Figures S10–S12 present the ^1^H NMR spectra of the raw materials and the three different types of MCs. The ^1^H NMR spectra of the MCs exhibit a perfect merging of the spectra of the raw materials. It is also relevant to mention that the isocyanurate ring from the HDI-polymer causes the split of the triplet at 3.30 ppm, assigned to the -CH_2_- next to the isocyanate groups, and that the iminooxadiazinedione ring has two triplets at 3.35 ppm and 3.82 ppm corresponding to the two protons next to the imine group, which are in different planes. No extra peaks or changes in the multiplicity or chemical shifts in the MCs’ spectra occurred, which indicates that significant isocyanate polymerisation did not occur during the emulsion or filtration stages.

The FTIR spectra of the MCs (Figure 5D) confirm the isocyanate encapsulation by the presence of a peak around 2260 cm^−1^. Peaks associated with the iminooxadiazinedione and isocyanurate ring (1788 cm^−1^ and 1682 cm^−1^) are evident for the trimer and polymer, and there is no indication of peaks related to the formation of PUa.

**Figure 6:**
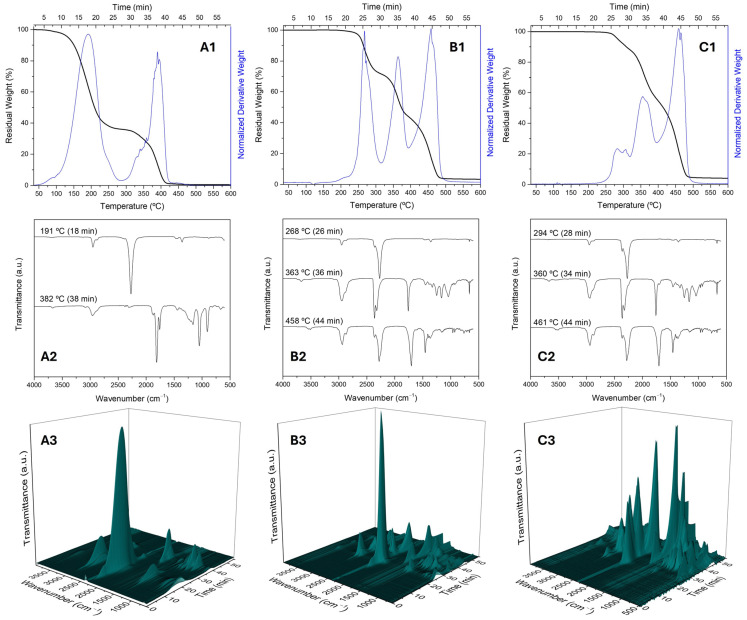
TGA-FTIR analysis of MCs-Monomer (**A1**–**A3**) showing TGA in nitrogen, FTIR curves measured at 191 °C and 382 °C, and 3D images of FTIR in nitrogen environment; MCs-Trimer (**B1**–**B3**) showing TGA in nitrogen, FTIR curves measured at 268 °C, 363 °C, and 458 °C, and 3D images of FTIR in nitrogen environment; MCs-Polymer (**C1**–**C3**) showing TGA in nitrogen, FTIR curves measured at 294 °C, 360 °C, and 461 °C, and 3D images of FTIR in nitrogen environment.

The isocyanate trimer and polymer are more difficult to quantify by TGA due to the multiple degradation stages occurring between 190 °C and 515 °C (Figure 6). Consequently, a precise evaluation of the MCs’ payload and PUa formation is complicated. Nonetheless, by comparing the peak ratios of the individual isocyanates with those of the MCs, it is possible to infer that very little polyurea was formed. The analyses using thermogravimetry coupled with Fourier transform infrared spectroscopy (TGA-FTIR), as depicted in Figure 6, allow for detailed information on the thermal decomposition of materials and the identification of gases released during the heating process. The continuous online identification of evolved gases, without intermediate trapping or isolation, prevents gas condensation and avoids side reactions before detection and FTIR characterisation. The analysis of the MCs-Monomer indicates that the first thermal event is due to the volatilisation of HDI, as the gas spectrum at 191 °C is identical to that of pure HDI. In contrast, the thermal event associated with the degradation of PBS (T_max_ at 382 °C) shows the presence of various gaseous components, which, as described by Lu et al. [61], result from the degradation of the polyester into compounds such as succinic acid, other carboxylic acids, alkenyl-terminated chain oligomers, and, in a later phase, into dihydrofuran-2,5-dione (anhydride) through the dehydration of succinic acid. The key FTIR bands confirming these functional groups are observed at 1875 cm^−1^, 1810 cm^−1^, 1760 cm^−1^, and 1050 cm^−1^, corresponding to the C=O and C-O-C groups of anhydrides and carboxylic acids, as well as at 3088 cm^−1^, 1640 cm^−1^, and 910 cm^−1^, associated with alkene groups (C-H stretching, C=C stretching, and C-H out-of-plane bending).

The thermograms of both MCs-Trimer and MCs-Polymer show three distinct stages of significant mass loss, with the FTIR spectra of the gases from these thermal events being remarkably similar. However, for MCs-Trimer, the mass loss in the first zone (between 175 °C and 325 °C) is more pronounced, leading to the conclusion that the isocyanurate ring is thermally more robust than the iminooxadiazinedione ring. The FTIR analysis of the gases reveals that, during this phase, there is a release of aliphatic isocyanates, confirmed by the band at 2270 cm^−1^ (N=C=O stretching) and the region 3025–2805 cm^−1^ related to C-H stretching, as well as some release of CO_2_ (doublet at 2360 and 2315 cm^−1^, partially hidden by the NCO band). The second stage of thermal loss, with a T_max_ around 360 °C, is associated with the degradation of the polymer. However, there is also a significant release of CO_2_, evidenced by a strong doublet at 2360 and 2315 cm^−1^ and a sharp peak at 668 cm^−1^, which is related to the degradation of isocyanates. It is important to note that the gas mixture resulting from the degradation of PBS is simpler in the MCs-Trimer and MCs-Polymer samples compared to PBS alone or the MCs-Monomer (Figure S13). The signal assigned to the C=O functional group is sharper (1760 cm^−1^), indicating the presence of anhydrides, and there are no longer other signals related to carbonyl or alkene groups.

The presence of a peak at 1699 cm^−1^ and the band around 3670 cm^−1^ in the MCs-Polymer indicates the release of cyanuric acid, which results from the loss of aliphatic isocyanate groups from the initial isocyanurate ring. The formation of components with hydroxyl and amine groups from the degradation of both the PBS and the isocyanates may lead to reorganisation and reactions with free isocyanate groups, causing the differences in the spectral profiles observed for the MCs-Trimer and MCs-Polymer compared to the raw materials or the MCs-Monomer. The third degradation stage occurred in the range between 400 and 520 °C and is characterised by the band associated with the stretching vibration of isocyanates (2270 cm^−1^), which should be aliphatic due to the presence of bands at 2940/2870 cm^−1^ corresponding to the CH_2_ deformation and CH_2_ stretching vibrations, as well as at 1453 cm^−1^ and 1360 cm^−1^ (bending vibrations of the C–H bonds). The peak at 1704 cm⁻¹ indicates the presence of cyanuric acid formed due to the elimination of the respective alkyl isocyanate, which, as Passauer indicated [62], can decompose into isocyanic acid, contributing to the broadening of the isocyanate band. The band at 3529 cm^−1^ should be assigned to the amine groups from cyanuric acid or from the aminohexyl isocyanurate by-product, which should also be considered in this type of degradation [62]. Additionally, CO_2_ was also detected.

**Figure 7:**
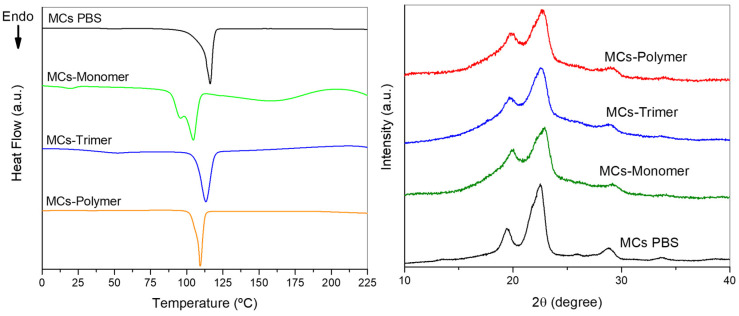
Thermograms (on the **left**) and diffractograms (on the **right**) of the MCs, with and without isocyanate.

The results from DSC (Figure 7) indicate that the melting behaviour of PBS shifts with the incorporation of isocyanate, leading to a decrease in the melting point. MCs-Trimer (Tm = 113 °C) and MCs-Polymer (Tm= 109 °C) show a slight reduction compared to the sample without isocyanate (Tm = 116 °C). MCs-Monomer, unlike the other two MCs, presents two distinct peaks of fusion for PBS. This means that two distinct types of crystalline PBS emerge due to HDI acting as a plasticiser to PBS in the MCs. HDI is of low viscosity and is small enough to easily diffuse through the PBS chains, meaning it can stay in the interstices of the crystal structure of the PBS, which decreases the intermolecular forces between chains and originates two distinct melting peaks in the DSC. The first one (at a lower melting temperature) is related to the crystalline polymer with a higher amount of HDI, and the second peak is the polymer with a smaller amount of isocyanate in its structure. This is proof of the matrix-type structure of the MCs, where some of the HDI was not able to diffuse out of the polymer-rich phase and became part of the crystalline structure of the polymer. Due to their higher molecular weight, the other two isocyanates cannot have the same effect as HDI, but still influence the melting point by hampering the crystallisation of PBS and the small decrease in the melting point. Thus, they are mostly present in the amorphous phase, which is not visible in the thermogram, but only by studying changes in the glass transition temperature of the polymer.

XRD was performed to understand whether any structural changes would occur during the microencapsulation process, such as changes in crystallinity, the influence of the isocyanate in the crystallinity, and the presence of PUa. The diffractograms (Figure 7) show that the MCs are semi-crystalline. PBS has a monoclinic α-form unit cell with the diffraction peaks of the crystal planes appearing at 19.5° (020), 22.5° (110), and 28.8° (111) [63]. When introducing the isocyanates in the MCs, a decrease in the intensity of the peaks also occurs due to the presence of isocyanate, which is in an amorphous state. The peaks are not as sharp, suggesting that the presence of isocyanate slightly hinders the crystallisation of PBS, broadening the peak. No major shifts in the position of the peaks occur, suggesting no major modifications in the lattice parameters of crystalline PBS. The presence of PUa would be noticed by a peak around 11.5° (Figure S14) [64]; since the diffractograms do not show such a peak or any significant modification of the signals attributed to PBS, we can confirm that there is no significant PUa in the MCs.

The percentage of encapsulated isocyanate was studied by NMR, and Table 1 displays the payload values. The NMR spectra are present in Figures S15–S17. All three isocyanates were successfully encapsulated in a PBS matrix, with high encapsulation values ranging from 67 wt% to 70 wt%. This indicates that the encapsulation degrees for the three isocyanate species are quite similar and not influenced by their individual physical or chemical properties.

**Figure 8:**
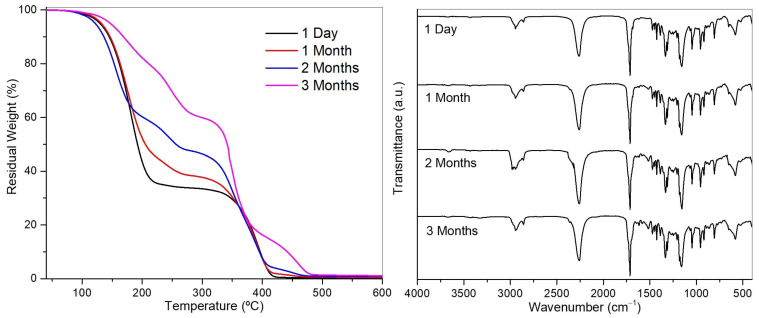
TGA (**left**) and FTIR spectra (**right**) of the MCs-Monomer stored for three months.

The MCs’ stability under 35% to 60% relative humidity at room temperature (15–25 °C) was monitored over 1, 2, and 3 months using FTIR, TGA, and NMR. As shown in Figure 8, the functional group of isocyanates (NCO group) is significantly preserved (FTIR spectra) over time, indicating that around 40 wt% of the MCs still corresponds to functional isocyanate species rather than polyurea over three months. However, as observed from the TGA curve profile and confirmed by NMR, by the third month, only a small percentage (less than 10 wt%) consists of the initial monomeric HDI, with the remaining NCO-containing species are likely to be oligomeric or polymeric derivatives of the monomeric HDI.

For the MCs-Trimer and MCs-Polymer, the TGA and FTIR analyses (Figures S18 and S19) show a faster decrease in isocyanate content and a more pronounced increase in PUa formation compared to HDI. After one month, the trimer retains 64 wt%, whereas the polymer shows a higher loss (54 wt%). However, due to the higher reactivity of the trimer [60], its percentage becomes almost negligible after 2 months, while the polymer only shows an isocyanate content below 10 wt% after 3 months. The fact that HDI, a more reactive isocyanate than the other two aliphatic isocyanates, does not convert as easily to PUa may be due to its integration into the polymer’s crystal structure, which makes its reaction with water more difficult.

**Figure 9:**
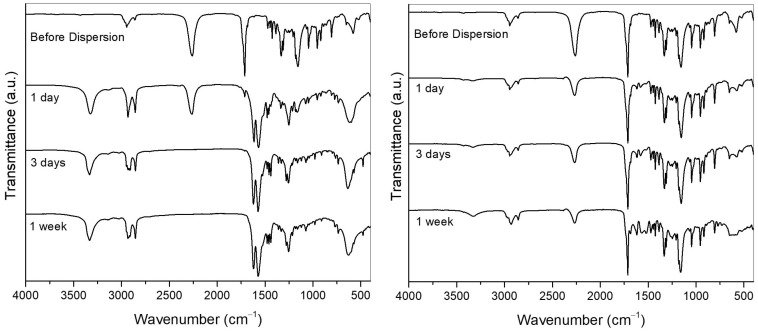
FTIR spectra of the MCs-Monomer exposed to water (**left**) and hexane (**right**) within a time span of one week.

When the MCs are applied in the adhesive, they come into contact with solvents. These solvents can be aliphatic hydrocarbons, like ketones, and esters [65] or water in the case of water-based adhesives. Therefore, the behaviour of the MCs and the permeability of their polymeric shell in different solvents were investigated. For this, 0.1 g of MCs was dispersed in 5 mL of different solvents (water, acetone, hexane, and ethyl acetate) and characterised after 1 day, 3 days, and 1 week in the solvent. As shown by the spectra in Figure 9 and Figures S20–S30, the MCs are permeable to the solvents, and their behaviour differs between organic solvents and water. The organic solvents promoted the leaching of the isocyanates. On the other hand, in water, PUa formation occurred inside the MCs. This is evident from the appearance of peaks corresponding to PUa in the FTIR (bands at 1617 and 1580 cm^−1^) for the MCs dispersed in water and the lack of these signals for the MCs dispersed in organic solvents. After one day, the MCs in water had the highest isocyanate content, and among the organic solvents, hexane showed the slowest leaching rate.

After exposing the MCs to the solvents, SEM images were taken, showing that the MCs’ morphology remained unchanged, with no visible PUa formation, supporting the hypothesis that PUa forms only inside the MCs when dispersed in water and no burst of the MCs occurred when exposed to the other solvents (Figure S31). This permeability feature is similar to what has been published for other MCs produced by solvent evaporation with other polyesters [39,54]. Although matrix-type MCs were expected to be more resistant than the core-shell MCs, a direct comparison might lead to some misperceptions due to their smaller diameters. This study also confirms that the release of isocyanate is highly efficient during the adhesive joint preparation, specifically at the stage of mixing MCs with the base adhesive component, and does not require the tearing or melting of the MC walls. As a result, it proves to be highly effective for applications where immediate release and crosslinking are needed, such as in footwear adhesives.

### 3.3. Adhesive Joint Characterisation

Adhesive formulations were prepared by blending either MCs or non-encapsulated isocyanates with a polyurethane-based adhesive (Plastik 6275). Peel and creep tests were performed on the test specimens following their preparation (as detailed in the materials and methods section) and after 3 days of curing. The FTIR analysis of the adhesive joint after curing (Figure S32) shows that all the isocyanate has reacted, as there is no signal at 2270 cm⁻¹ (N=C=O stretching).

**Figure 10:**
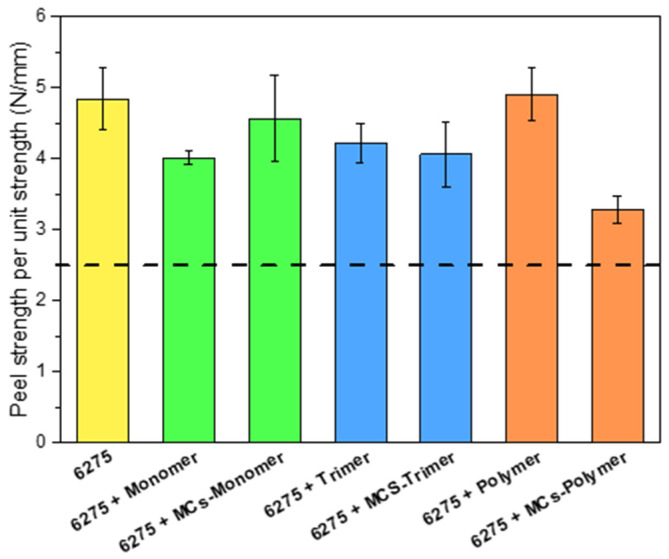
Peel strength of the adhesive joints with and without MCs; Dashed line indicates the minimum necessary to be applied in footwear.

The peel strength (Figure 10) is an important parameter for assessing whether the encapsulation of isocyanate impacts the joints’ mechanical properties. The minimum required strength so that the adhesive joint can be applied in the footwear industry is 2.5 N/mm [66]. As shown in Figure 10 and Table S4, the peel strength test results are above the threshold value, indicating the suitability of the adhesive for footwear applications. Regarding the non-encapsulated isocyanates, the monomer has a higher decrease in strength, which could be justified by the high volatility of this compound (0.05 mmHg at 25 °C), which causes isocyanate lost during the drying and reactivation step. The trimer and the polymer present a slightly higher strength due to the crosslinking that the several isocyanate groups provide. An increase is only observed with MCs-Monomer, likely due to the high volatility of HDI. In this case, encapsulating the isocyanate helps prevent its premature release. The reduction in peel strength was more pronounced in the formulation with MCs-Polymer, which can be attributed to its greater tendency to form aggregates and lumps, potentially acting as defects in the joint. Despite the reduction in peel strength when MCs-Trimer and MCs-Polymer were used, all adhesive joints exceeded 3 N/mm.

One of the main functions of isocyanate in the upper-sole bond sort of adhesive is to enhance the crosslinking within the adhesive joint, thus improving the heat resistance. The creep test enables the test of creep strength, a parameter directly linked to the adhesive’s ability to withstand temperature variations under constant stress without structural damage. This attribute is particularly important because footwear frequently experiences significant temperature variations during storage, exposure, and usage.

As stated in Table 2, the specimens with only Plastik 6275 have low-temperature resistance, with the adhesive starting to open at 60 °C and fully opening at 70 °C. As expected, the addition of isocyanates enhances the temperature resistance of the adhesive bond. Except for the sample with the monomer, the samples with non-encapsulated isocyanates performed excellently in the creep test, showing no signs of opening up to at least 90 °C. Similar to the peel test, the lower performance of the “6275 + Monomer” sample is attributed to the higher volatility of this isocyanate.

The comparison between samples with MCs and those with non-encapsulated isocyanates shows different features. Comparing the adhesives with monomer, those with MCs performed better. For the trimer, both encapsulated and non-encapsulated samples yielded equally excellent results. The specimens with formulations of “6275 + MCs-Polymer” showed the poorest results, as they only withstood temperatures up to 70 °C. This result is directly related to the formation of lumps in the formulation, which is difficult to mitigate during application (Figure S33). Since only the MCs-Polymer formed lumps with the adhesive, we attribute this issue solely to the isocyanate, given that the MCs-Trimer has a similar morphology and similar diameters. For MCs with HDI, displacement began only at 80 °C, indicating that encapsulation effectively protects the isocyanate from early evaporation or degradation, leading to improved results even when compared to non-encapsulated isocyanate, which is consistent with the outcomes of the peel tests. The specimens with “6275 + MCs-Trimer” formulations proved even more resistant, showing no opening across any temperature range. This outstanding performance places this formulation on par with the best reported so far [39,54].

Additionally, these results reveal that matrix-type MCs do not negatively influence the mechanical properties of the joint or the release of isocyanate in the formulation, performing as well as or better than similar core-shell MCs [39,40,54].

## 4. Conclusions

This study demonstrates the successful production of MCs using a bio-based and biodegradable shell material (bio-PBS) loaded with three different aliphatic isocyanates. This allows for the safer production of adhesive joints, without direct contact with isocyanate species, which represents significant benefits from a health security perspective. These new MCs exhibit a regular spherical shape, no holes or fractures, median diameter sizes between 34 and 65 µm, and the high loading (up to 70 wt%) of isocyanate.

The production parameters have an important impact on the shape and size of the final MCs, with the amount of PVA having a greater influence on size distribution than the amount of isocyanate. The structure of the MCs was studied by XRD and DSC, revealing the incorporation of HDI species within the crystalline structure of PBS, which led to a change in the crystallinity of PBS and a decrease in the melting point of PBS. The bio-PBS MCs’ shelf life in a moderately humid environment aligns with other published MCs, with the MCs-Monomer exhibiting the least loss in terms of functional NCO groups. The permeability of the bio-PBS matrix allows for an optimal release of isocyanate when the MCs are mixed with the base adhesive component, promoting good crosslinking within the adhesive joint, together with a negligible effect on the polymeric wall of the MCs in the adhesive’s performance.

Besides the improved safety of the process, these new MCs showed a positive impact in the adhesive joints when compared to the same non-encapsulated isocyanates. The peel strength of the adhesives with the encapsulated isocyanate presents values above the threshold imposed by the footwear industry (≥2.5 N/mm). The creep tests show that the introduction and encapsulation of isocyanates enhance the properties of the joint. The adhesive joints made from the mixture of adhesive with MCs-Trimer were by far the best, with a peel strength greater than 4 N/mm and temperature resistance above 90 °C. This result is particularly noteworthy as it represents the first example of isocyanate-trimer encapsulation based on an iminooxadiazinedione ring, showcasing this relatively new and unexplored HDI-derived trimer as an effective crosslinker for adhesive applications. Additionally, this study demonstrates that matrix-type bio-based MCs can perform as well as or better than core-shell MCs in adhesives.

## Figures and Tables

**Table 1:** Quantification of the isocyanates by NMR.

Samples	1 Day	1 Month	2 Months	3 Months
MCs-Monomer	67 wt%	56 wt%	42 wt%	<10 wt%
MCs-Trimer	70 wt%	64 wt%	<10 wt%	<10 wt%
MCs-Polymer	69 wt%	54 wt%	14 wt%	<10 wt%

**Table 2:** Results for the creep strength of the adhesive joints with and without encapsulated isocyanates. “Full opening” corresponds to a minimum detachment of 10 cm.

Sample	60 °C	70 °C	80 °C	90 °C
6275	1.0 ± 0.3 cm	Full opening	Full opening	Full opening
6275 + Monomer	0.33 ± 0.05 cm	1.2 ± 0.7 cm	9.3 ± 1.0 cm	Full opening
6275 + MCs-Monomer	0 ± 0 cm	0 ± 0 cm	0 ± 0 cm	Full opening
6275 + Trimer	0 ± 0 cm	0 ± 0 cm	0 ± 0 cm	0 ± 0 cm
6275 + MCs-Trimer	0 ± 0 cm	0 ± 0 cm	0 ± 0 cm	0 ± 0 cm
6275 + Polymer	0 ± 0 cm	0 ± 0 cm	0 ± 0 cm	0 ± 0 cm
6275 + MCs-Polymer	0 ± 0 cm	0.87 ± 0.05 cm	Full opening	Full opening

## Data Availability

Data are contained within the article and Supplementary Materials.

## Supplementary Materials

The following supporting information can be downloaded at: https://www.mdpi.com/article/10.3390/polym17020139/s1, Figure S1: ^1^H NMR spectra with the ratio of the signals obtained and mass of isocyanate; Figure S2: Calibration curve for the calculation of HDI-based polymer inside the MCs; Table S1: Statistical analysis regarding particle size distribution obtained with different amounts of emulsifier; Figure S3: Histograms of the size of the MCs changing the amount of emulsifier; Figure S4: FTIR Spectrum of PBS; Figure S5: FTIR of the encapsulated isocyanates; Figure S6: FTIR peaks (in red) related to the formation of PUa in the MCs; Figure S7: TGA and DTG of PBS and of the encapsulated isocyanates; Table S2: Statistical analysis regarding particle size distribution obtained with different quantities of isocyanate; Figure S8: Histograms of the size of the MCs changing the amount of HDI; Table S3: Statistical analysis regarding particle size distribution obtained with different isocyanates; Figure S9: Histograms of the size of the MCs changing the type of isocyanate encapsulated; Figure S10: ^1^H NMR spectra for PBS, monomer, and the obtained MCs; Figure S11: ^1^H NMR spectra for PBS, trimer, and the obtained MCs; Figure S12: ^1^H NMR spectra for PBS, polymer, and the obtained MCs; Figure S13: FTIR of the gas produced by PBS at 390 °C; Figure S14: Diffractogram of polyurea (PUa); Figure S15: 1H NMR spectra of MCs-Monomer (14.7 mg) + 4-chloro-3-methylphenol (7.8 mg) in CDCl_3_; Figure S16: ^1^H NMR spectra of MCs-Trimer (14.7 mg) + 4-chloro-3-methylphenol (8 mg) in CDCl_3_; Figure S17. ^1^H NMR spectra of MCs-Polymer (13.5 mg) + 4-chloro-3-methylphenol in CDCl_3_; Figure S18: FTIR spectra (top), TGA (bottom-left), and DTG (bottom-right) of the MCs-Trimer stored during the time span of three months; Figure S19: FTIR spectra (top), TGA (bottom-left), and DTG (bottom-right) of the MCs-Polymer stored during the time span of three months; Figure S20: TGA of MCs-Monomer when exposed to acetone (on the left) and hexane (on the right); Figure S21: FTIR (top-left), TGA (top-right), and DTG (bottom) of MCs-Monomer in acetone; Figure S22: FTIR (top-left), TGA (top-right), and DTG (bottom) of MCs-Monomer in hexane; Figure S23: FTIR (top-left), TGA (top-right), and DTG (bottom) MCs-Trimer in ethyl acetate; Figure S24: FTIR (top-left), TGA (top-right), and DTG (bottom) MCs-Trimer in water; Figure S25: FTIR (top-left), TGA (top-right), and DTG (bottom) MCs-Trimer in acetone; Figure S26: FTIR (top-left), TGA (top-right), and DTG (bottom) MCs-Trimer in hexane; Figure S27: FTIR (top-left), TGA (top-right), and DTG (bottom) MCs-Polymer in water; Figure S28: FTIR (top-left), TGA (top-right), and DTG (bottom) MCs-Polymer in acetone; Figure S29: FTIR (top-left), TGA (top-right), and DTG (bottom) MCs-Polymer in ethyl acetate; Figure S30: FTIR (top-left), TGA (top-right), and DTG (bottom) MCs-Polymer in hexane; Figure S31: MCs with isocyanate after exposure to solvents after 1 week in water (top) and hexane (bottom); Table S4: Results from the peel strength tests with and without encapsulated isocyanates; Figure S32: FTIR spectrum of the adhesive joint after 3 days of curing; Figure S33: Lump formation in the application of 6275 + MCs-Polymer in the substracts.
