# Supplementary material for: Bio-Microcapsules of Polybutylene Succinate (PBS) and Isocyanates: Towards Sustainable, Safer, and Efficient Adhesives
Source: Polymers (Basel). 2025 Jan 8;17(2):139. doi: 10.3390/polym17020139 (PMC11768415; doi:10.3390/polym17020139)
Supplement: Supplementary file 1 [file polymers-17-00139-s001.zip › polymers-3398699-supplementary.pdf]

Support Information

Bio-Microcapsules of Polybutylene Succinate (PBS) and Isocyanates: Towards Sustainable, Safer, and Efficient Adhesives

Lucas P. Marcelino [1], António Aguiar [1,*], Rui Galhano dos Santos [1], Isabel Pinho [2] and Ana C. Marques [1,*]

[1] CERENA-Centro de Recursos Naturais e Ambiente, Department of Chemical Engineering (DEQ), Instituto Superior Técnico, Universidade de Lisboa, Avenida Rovisco Pais, 1049-001 Lisboa, Portugal

[2] CIPADE S.A., Av. Primeiro de Maio 121, 3700-227 São João da Madeira, Portugal

* Correspondence: antonio.luis.aguiar@tecnico.ulisboa.pt (A.A.); ana.marques@tecnico.ulisboa.pt (A.C.M.)

Index

Figure S1. 1 H NMR spectra with the ratio of the signals obtained and mass of isocyanate. 3

Table S1. Statistical analysis regarding particle size distribution obtained with different amounts of emulsifier. 3

Figure S2. Calibration curve for the calculation of HDI-based polymer inside the MCs 3

Figure S3. Histograms of the size of the MCs changing the amount of PVA.........................4

Figure S4. FTIR Spectrum of PBS. 4

Figure S5. FTIR of the encapsulated isocyanates. 4

Figure S6. FTIR peaks (in red) related to the formation of PUa in the MCs. 5

Table S2. Statistical analysis regarding particle size distribution obtained with different quantities of isocyanate. 5

Figure S7. TGA and DTG of PBS and of the encapsulated isocyanates. 5

Table S3. Statistical analysis regarding particle size distribution obtained with different isocyanates........ 6

Figure S8. Histograms of the size of the MCs changing the amount of HDI..........................8

Figure S9. Histograms of the size of the MCs changing the type of isocyanate encapsulated. ... 6

Figure S10. 1H NMR spectra for PBS, monomer and the obtained MCs. 7

Figure S11. 1H NMR spectra for PBS, trimer and the obtained MCs. 7

Figure S12. 1H NMR spectra for PBS, polymer and the obtained MCs. 8

Figure S13. FTIR of the gas produced by PBS at 390ºC 8

Figure S14. Diffractogram of polyurea (PUa). 9

Figure S15. 1H NMR spectra of MCs-Monomer (14.7 mg) + 4-chloro-3-methylphenol (7.8 mg), in CDCl3. 9

Figure S16. 1H NMR spectra of MCs-Trimer (14.7 mg) + 4-chloro-3-methylphenol (8 mg), in CDCl3. 9

Figure S17. ^{1}H NMR spectra of MCs-Polymer (13.5 mg) + 4-chloro-3-methylphenol, in $CDCl_3$........11

Figure S18. FTIR spectra (top), TGA (bottom left) and DTG (bottom right) of the MCs-Trimer stored during the time span of three months. 10

Figure S19. FTIR spectra (top), TGA (bottom left) and DTG (bottom right) of the MCs-Polymer stored during the time span of three months. 11

Figure S20. TGA of MCs-Monomer when exposed to acetone (on the left) and hexane (on the right). 11

Figure S21. FTIR (top-left), TGA (top-right) and DTG (bottom) of MCs-Monomer in acetone.... 12

Figure S22. FTIR (top-left), TGA (top-right) and DTG (bottom) of MCs-Monomer in hexane..... 12

Figure S23.FTIR (top-left), TGA (top-right) and DTG (bottom) MCs-Trimer in ethyl acetate. 13

Figure S24. FTIR (top-left), TGA (top-rig ht) and DTG (bottom) MCs-Trimer in water. 13

Figure S25. FTIR (top-left), TGA (top-right) and DTG (bottom) MCs-Trimer in acetone............ 14

Figure S26. FTIR (top-left), TGA (top-right) and DTG (bottom) MCs-Trimer in hexane............. 14

Figure S27. FTIR (top-left), TGA (top-right) and DTG (bottom) MCs-Polymer in water. 15

Figure S28. FTIR (top-left), TGA (top-right) and DTG (bottom) MCs-Polymer in acetone.......... 15

Figure S29. FTIR (top-left), TGA (top-right) and DTG (bottom) MCs-Polymer in ethyl acetate. . 16

Figure S30. FTIR (top-left), TGA (top-right) and DTG (bottom) MCs-Polymer in hexane........... 16

Figure S31. MCs with isocyanate after exposure to solvents after 1 week in water (top) and hexane (bottom) 17

Figure S32. Lumps formation in the application of 6275 + MCs-Polymer in the substracts. 17

Table S4. Results from the peel strength tests with and without encapsulated isocyanates. 18

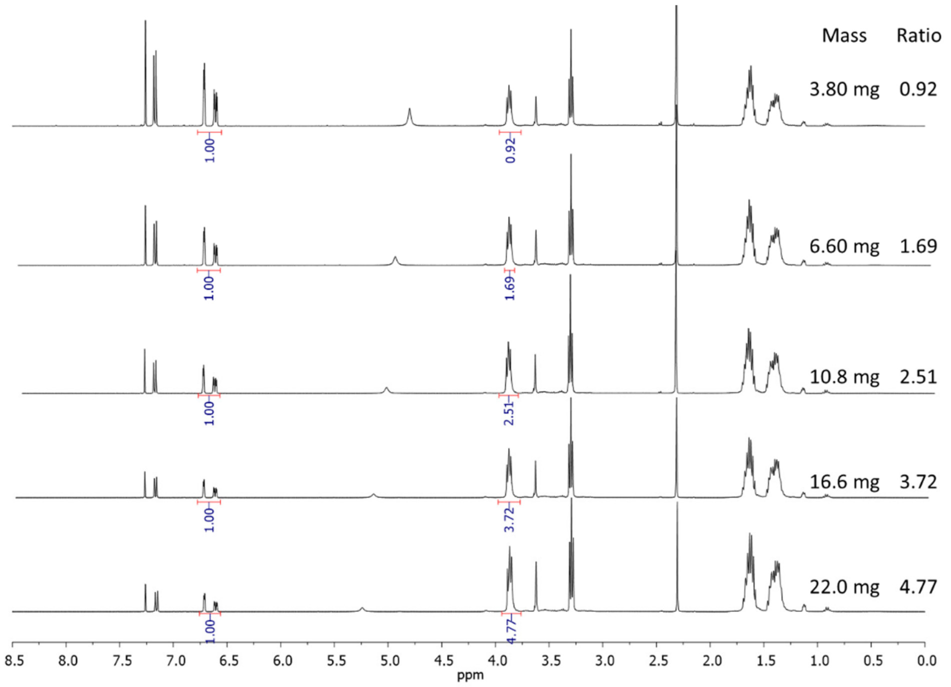

Figure S2. ^{1}H NMR spectra with the ratio of the signals obtained and mass of isocyanate.

Table S1. Statistical analysis regarding particle size distribution obtained with different amounts of emulsifier.

**Kruskal-Wallis**		
χ^2	DF	p
614	2	<0.001
**Dwass-Steel-Critchlow-Fligner**		
	W	p
1 wt% - 2 wt%	-26.5	<0.001
1 wt% - 3 wt%	-31.6	<0.001
2 wt% - 3 wt%	-15.3	<0.001

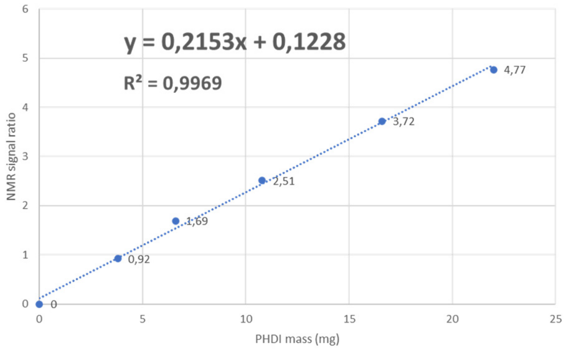

Figure S2. Calibration curve for the calculation of HDI-based polymer inside the MCs

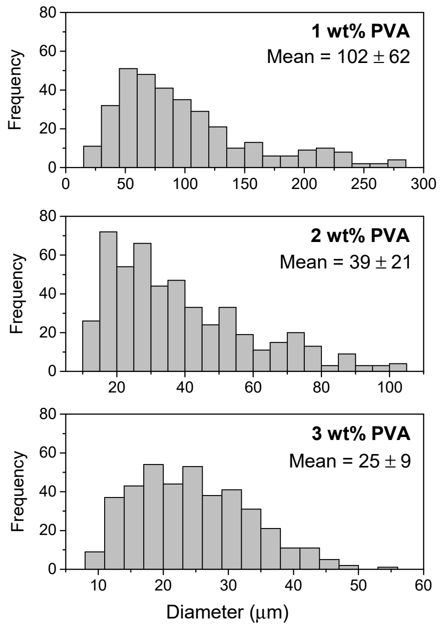

Figure S3. Histograms of the size of the MCs changing the amount of PVA.

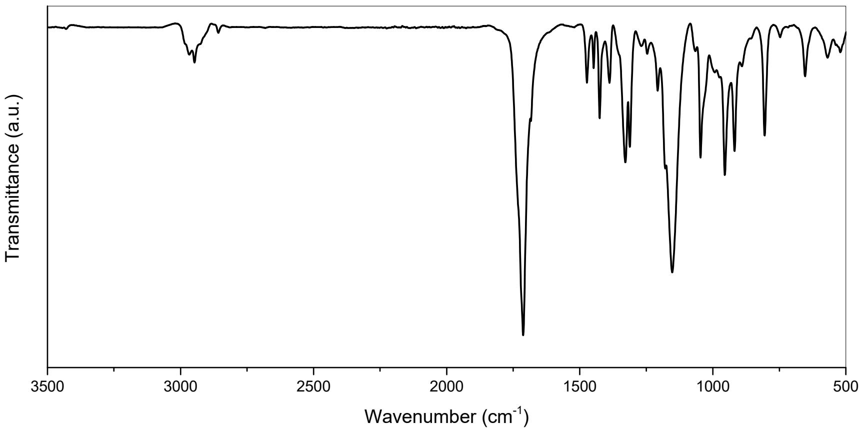

Figure S4. FTIR spectrum of PBS.

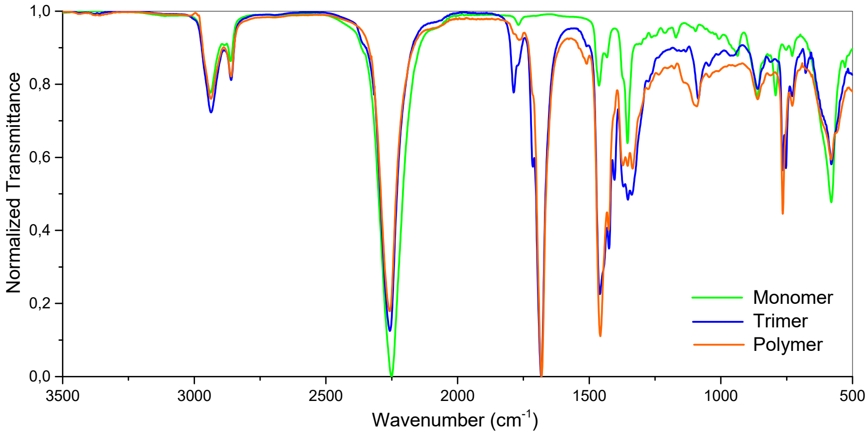

Figure S3. FTIR speactra of the encapsulated isocyanates.

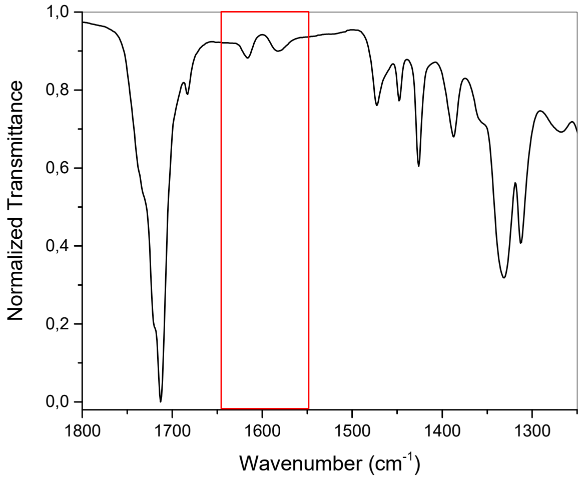

Figure S4. FTIR peaks (in red) related to the formation of PUa in the MCs.

Table S2. Statistical analysis regarding particle size distribution obtained with different quantities of isocyanate.

**Kruskal-Wallis**		
χ^2	DF	p
533	2	<0.001
**Dwass-Steel-Critchlow-Fligner**		
	W	p
8 wt% - 15 wt%	-13.75	<0.001
8 wt% - 20 wt%	26.97	<0.001
15 wt% - 20 wt%	28.78	<0.001

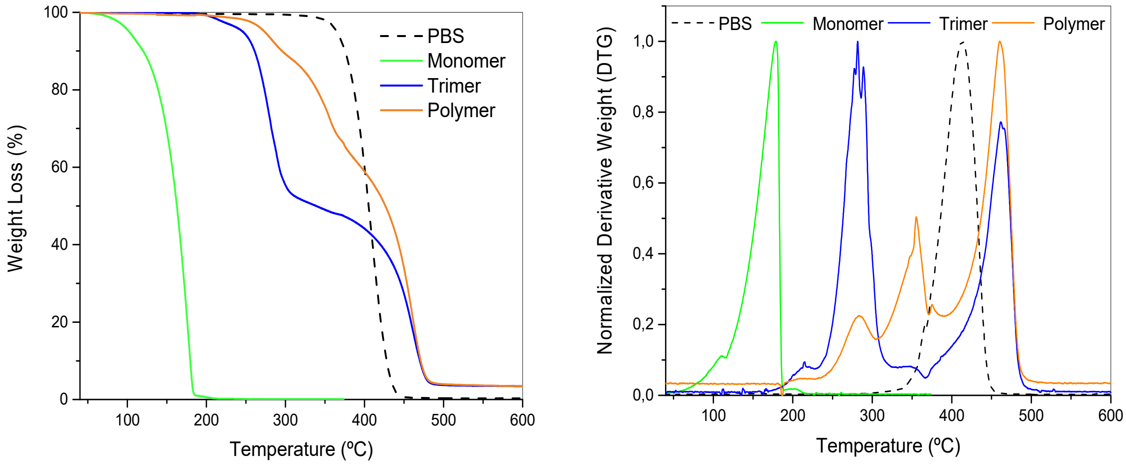

Figure S5. TGA and DTG of PBS and of the encapsulated isocyanates.

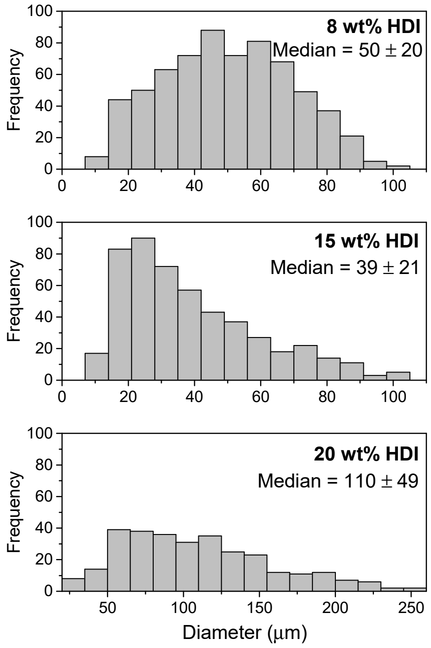

Figure S8. Histograms of the size of the MCs changing the amount of HDI.

Table S3. Statistical analysis regarding particle size distribution obtained with different isocyanates.

**Kruskal-Wallis**		
χ^2	DF	p
200	2	<0.001
**Dwass-Steel-Critchlow-Fligner**		
	W	p
Monomer-Trimer	15.77	<0.001
Monomer-Polymer	17.57	<0.001
Trimer-Polymer	-2.37	0.214

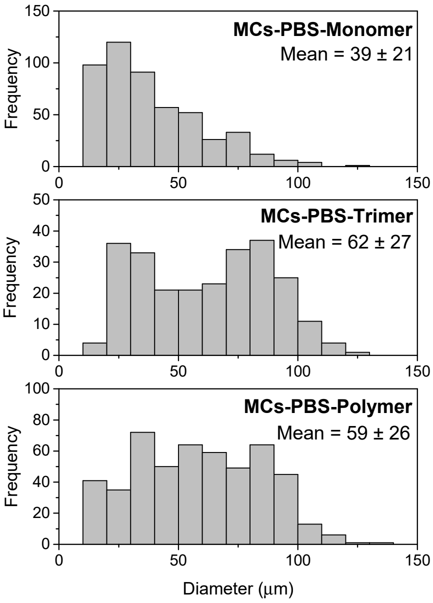

Figure S9. Histograms of the size of the MCs changing the type of isocyanate encapsulated.

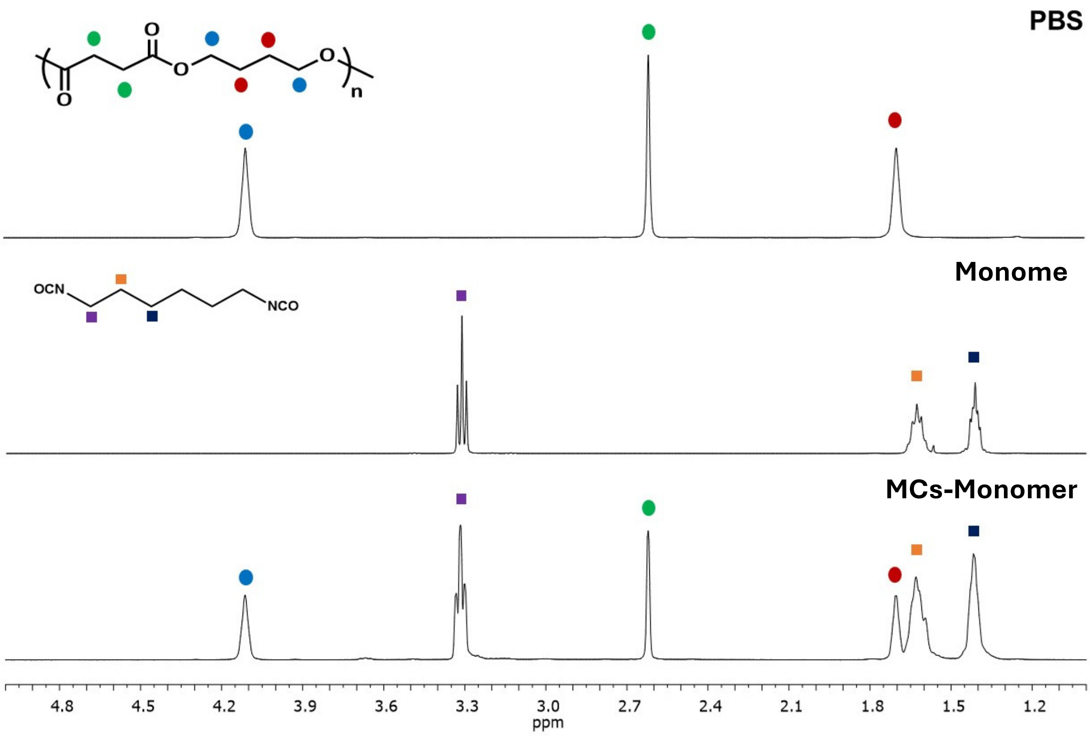

Figure S10. ^{1}H NMR spectra for PBS, monomer and the obtained MCs.

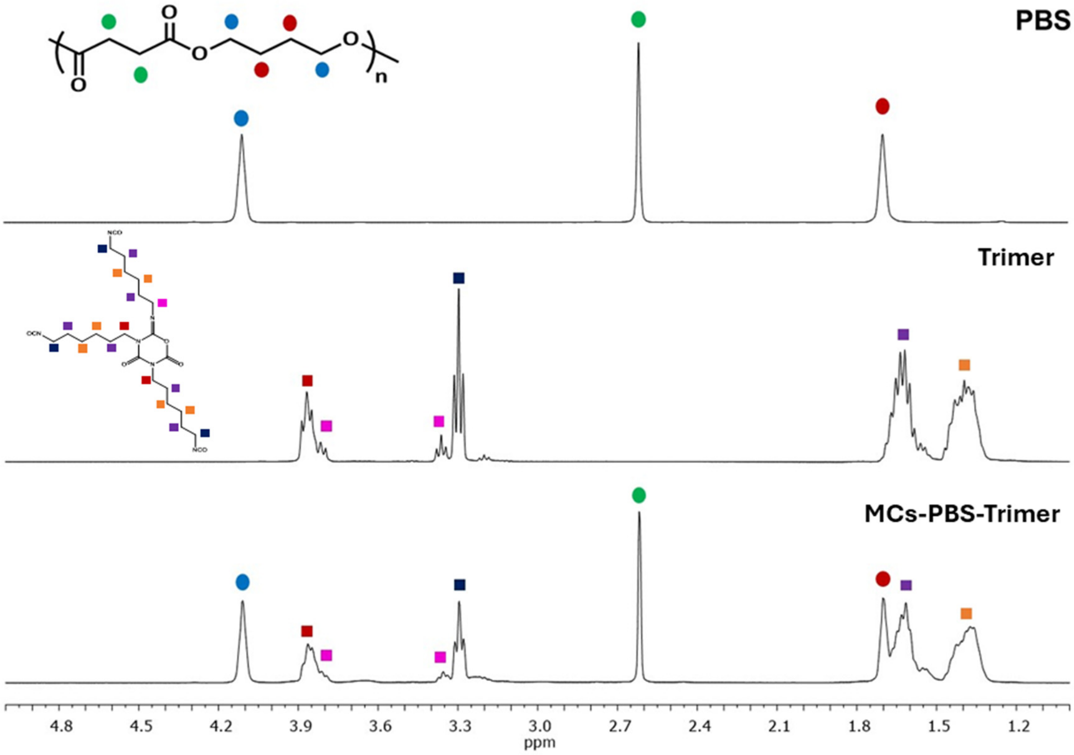

Figure S11. ^{1}H NMR spectra for PBS, trimer and the obtained MCs.

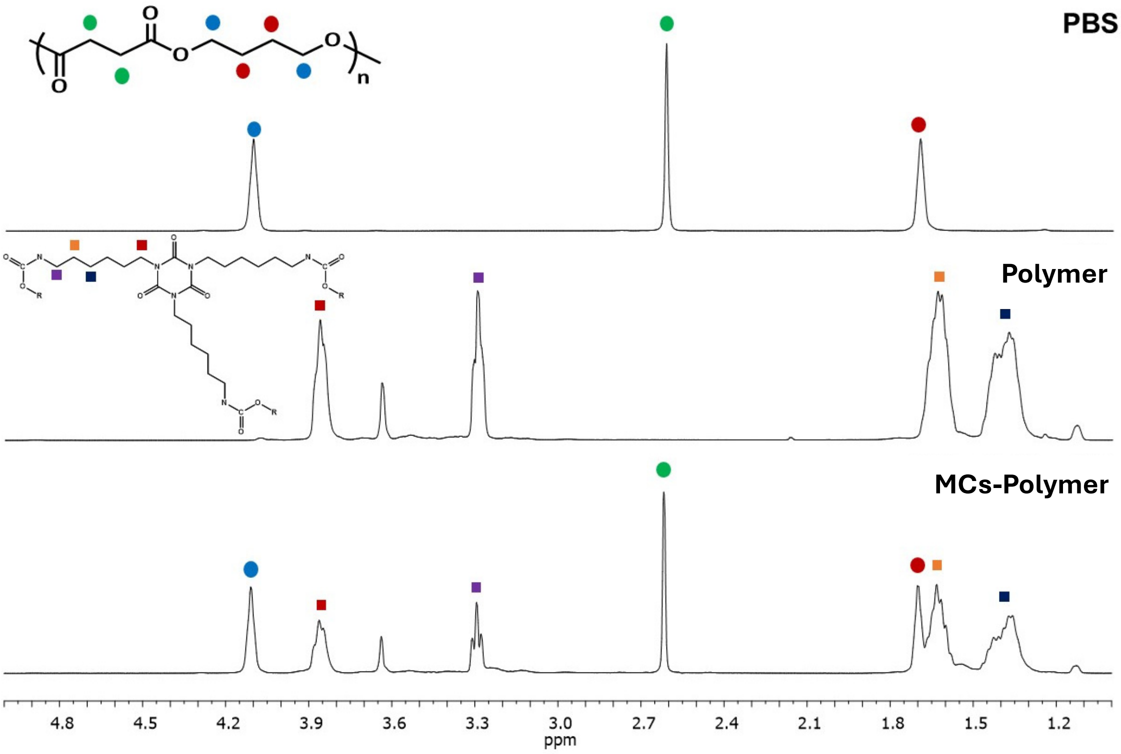

Figure S12. ^{1}H NMR spectra for PBS, polymer and the obtained MCs.

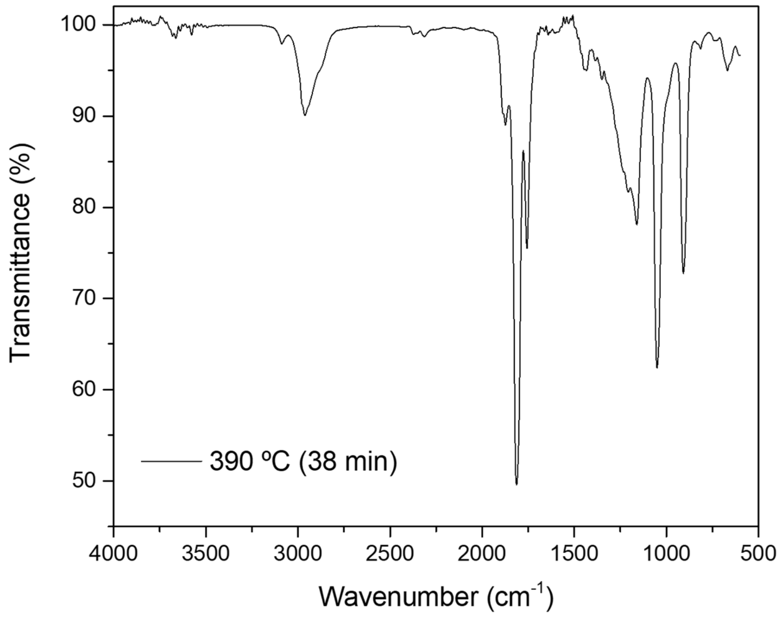

Figure S6. FTIR spectrum of the gas produced by PBS at 390ºC

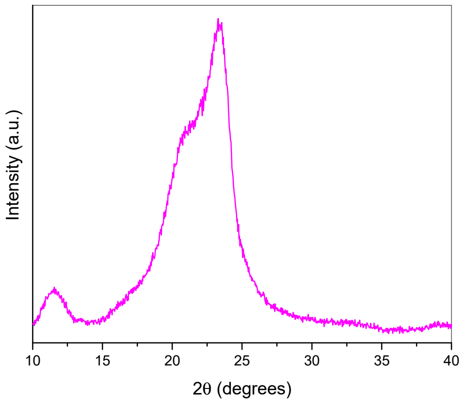

Figure S14. Diffractogram of polyurea (PUa).

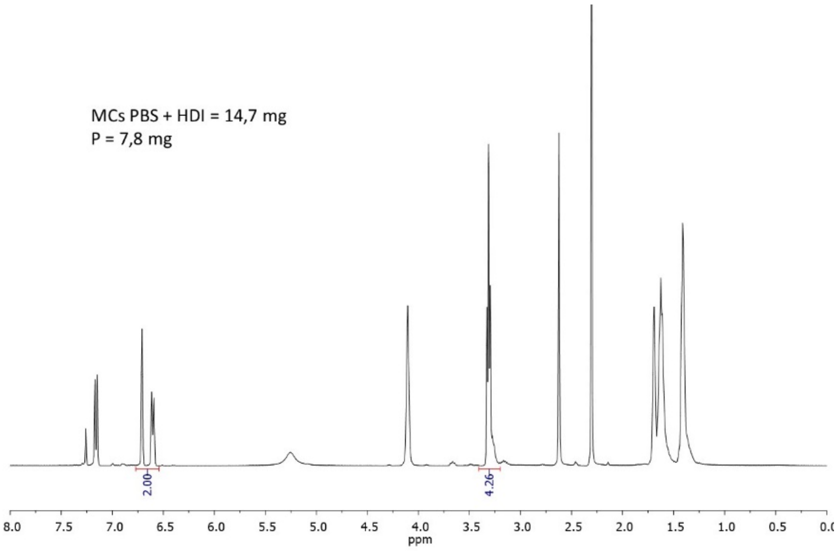

Figure S15. ^{1}H NMR spectra of MCs-Monomer (14.7 mg) + 4-chloro-3-methylphenol (7.8 mg), in $CDCl_3$.

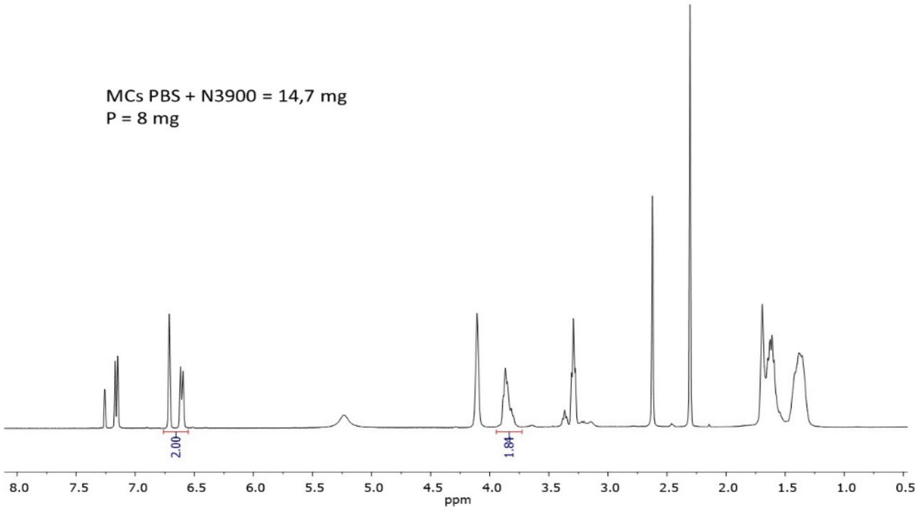

Figure S16. ^{1}H NMR spectra of MCs-Trimer (14.7 mg) + 4-chloro-3-methylphenol (8 mg), in $CDCl_3$.

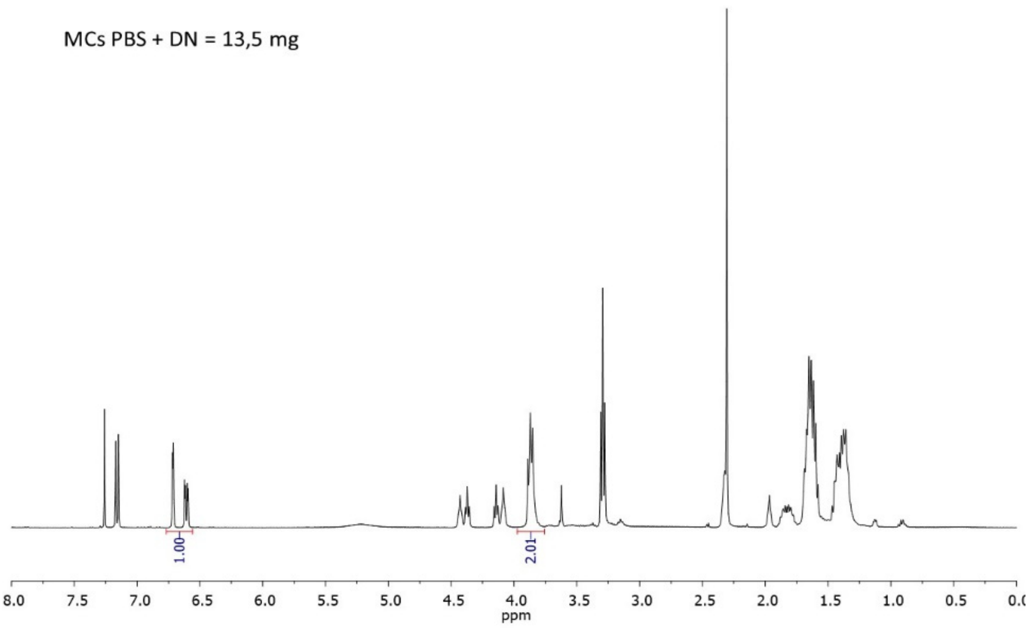

Figure S17. ^{1}H NMR spectra of MCs-Polymer (13.5 mg) + 4-chloro-3-methylphenol, in $CDCl_3$.

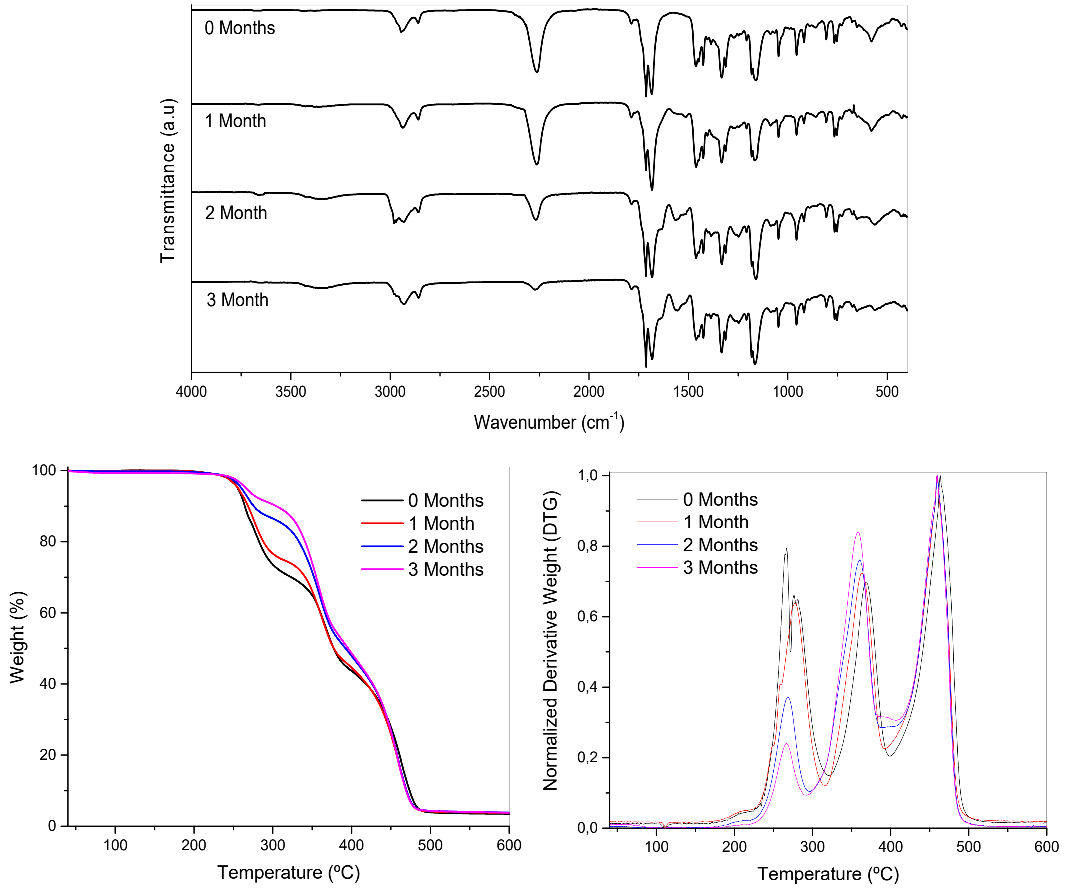

Figure S18. FTIR spectra (top), TGA (bottom left) and DTG (bottom right) of the MCs-Trimer stored during the time span of three months.

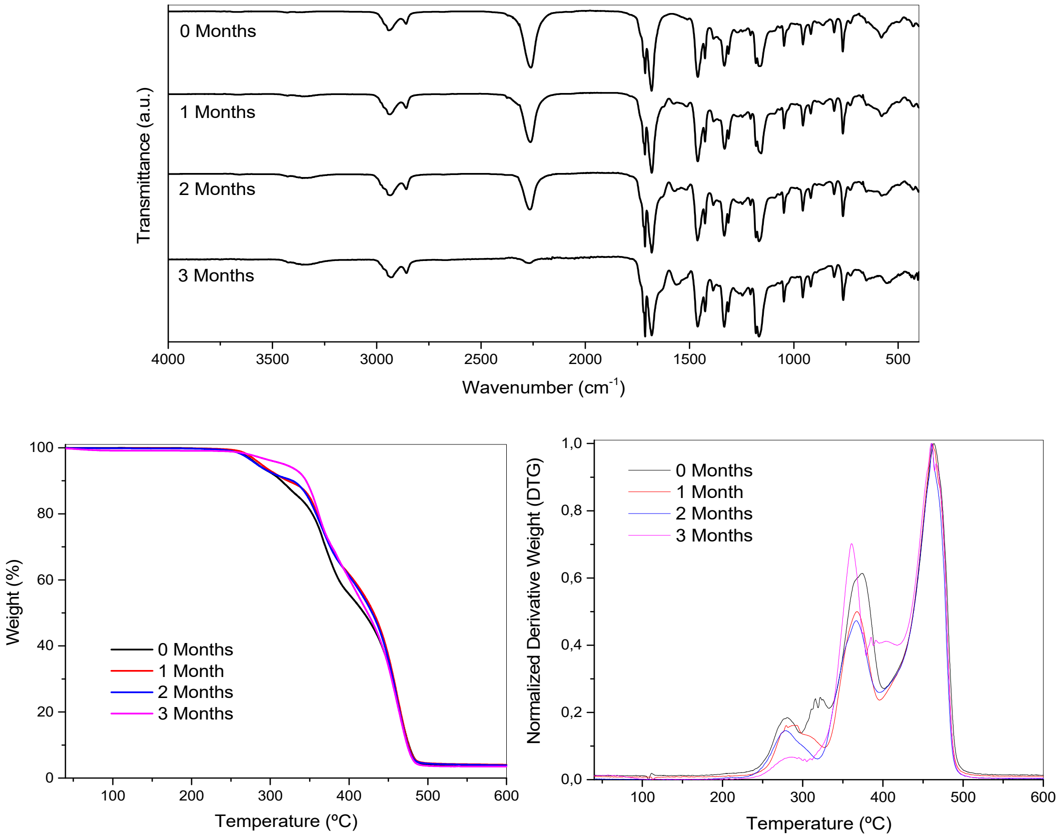

Figure S19. FTIR spectra (top), TGA (bottom left) and DTG (bottom right) of the MCs-Polymer stored during the time span of three months.

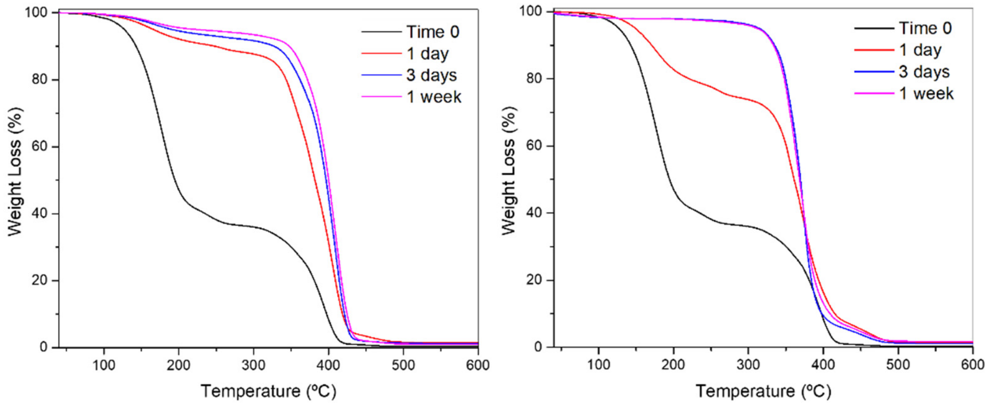

Figure S20. TGA of MCs-Monomer when exposed to acetone (on the left) and hexane (on the right).

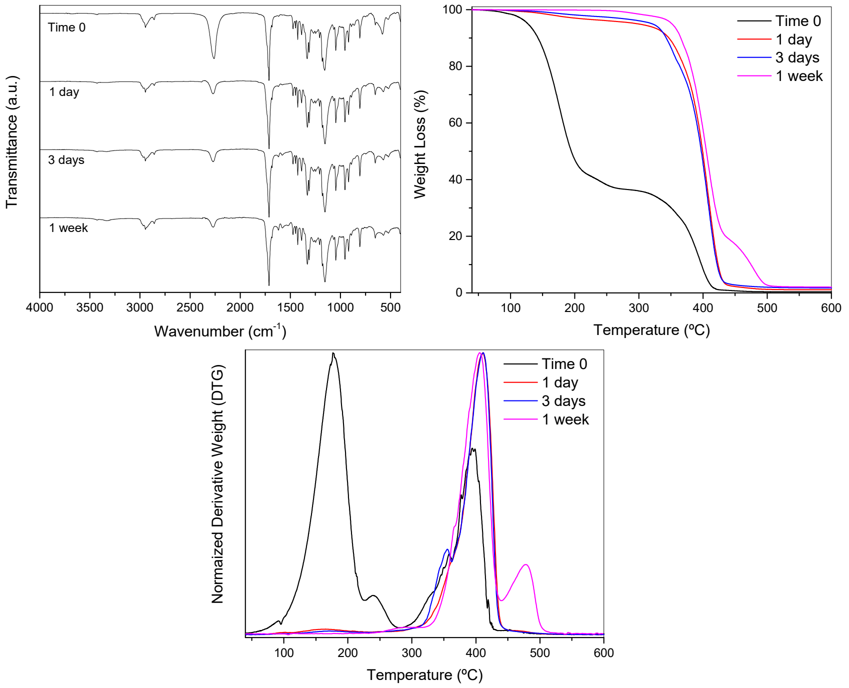

Figure S21. FTIR (top-left), TGA (top-right) and DTG (bottom) of MCs-Monomer in acetone.

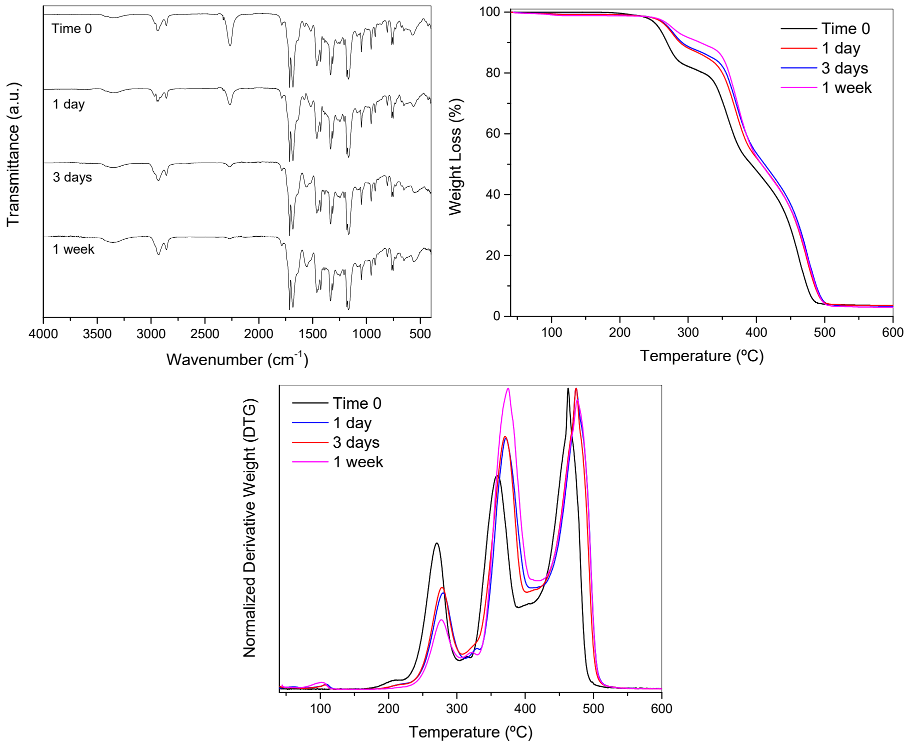

Figure S22. FTIR (top-left), TGA (top-right) and DTG (bottom) of MCs-Monomer in hexane.

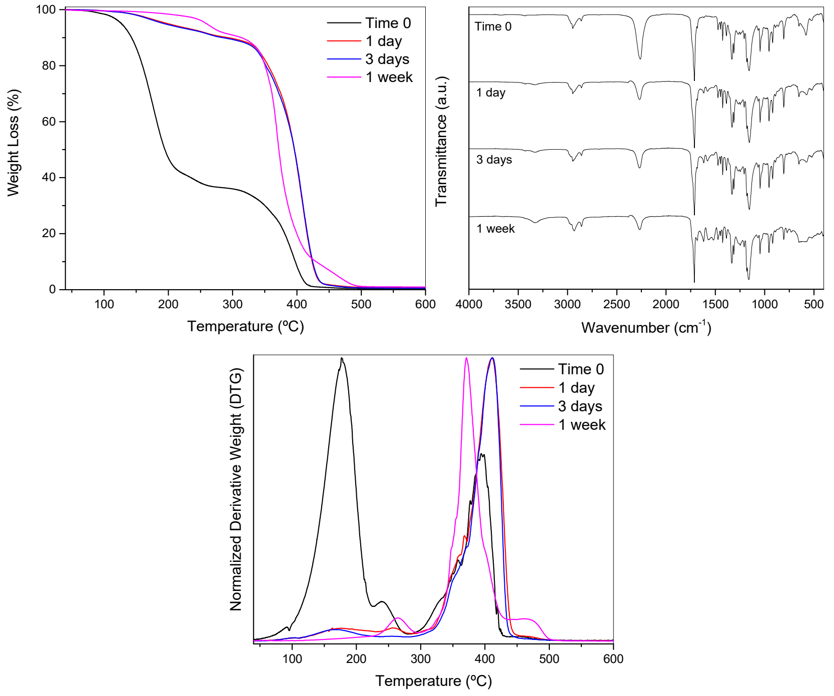

Figure S23.FTIR (top-left), TGA (top-right) and DTG (bottom) MCs-Trimer in ethyl acetate.

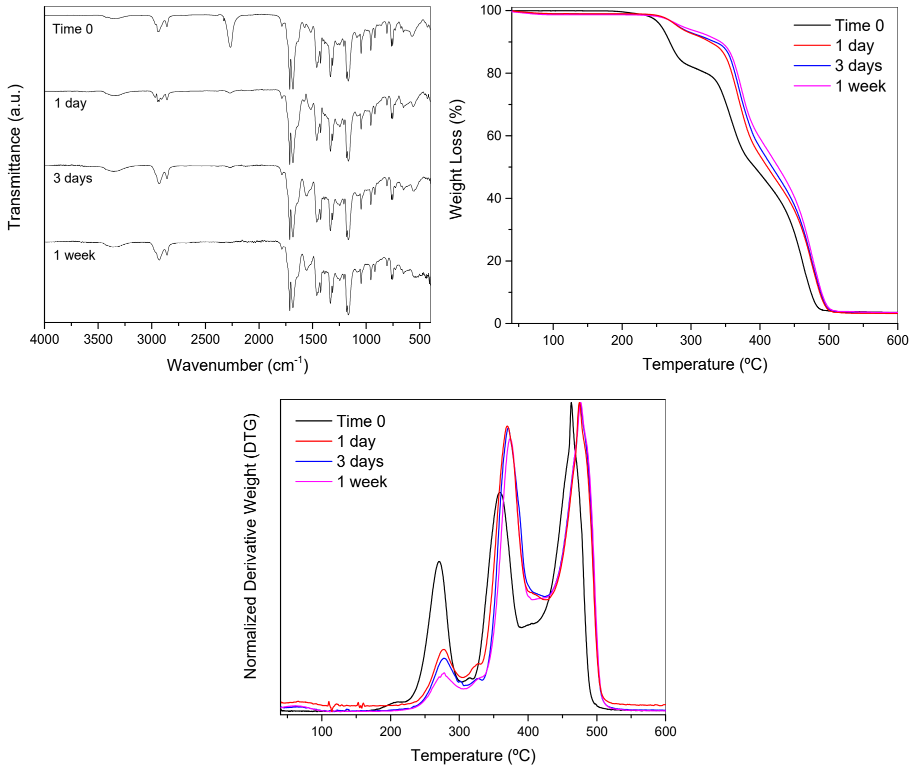

Figure S24. FTIR (top-left), TGA (top-rig ht) and DTG (bottom) MCs-Trimer in water.

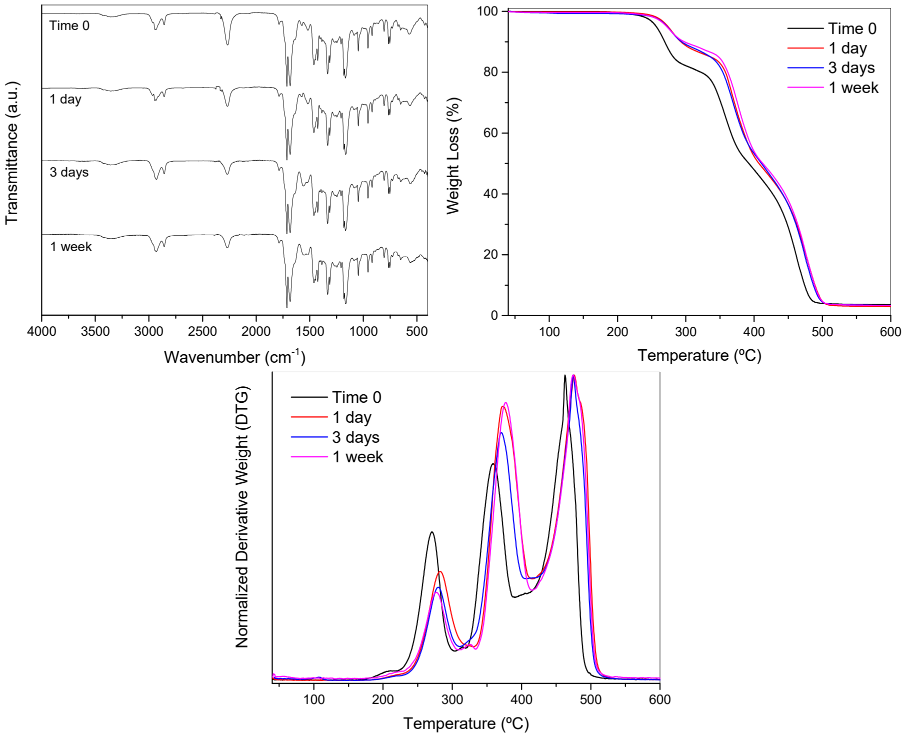

Figure S25. FTIR (top-left), TGA (top-right) and DTG (bottom) MCs-Trimer in acetone.

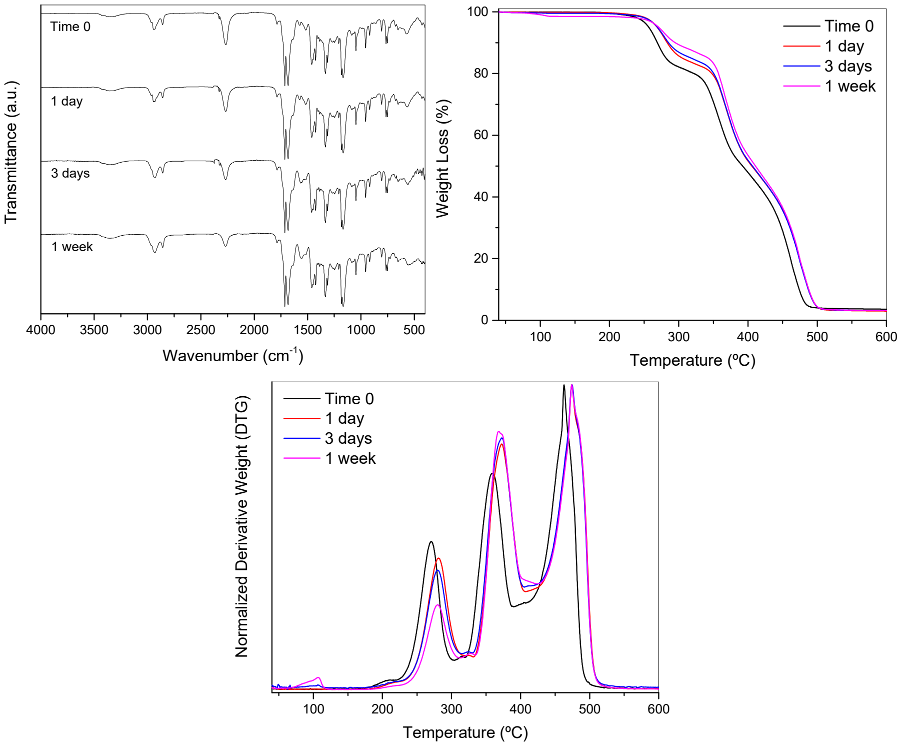

Figure S26. FTIR (top-left), TGA (top-right) and DTG (bottom) MCs-Trimer in hexane.

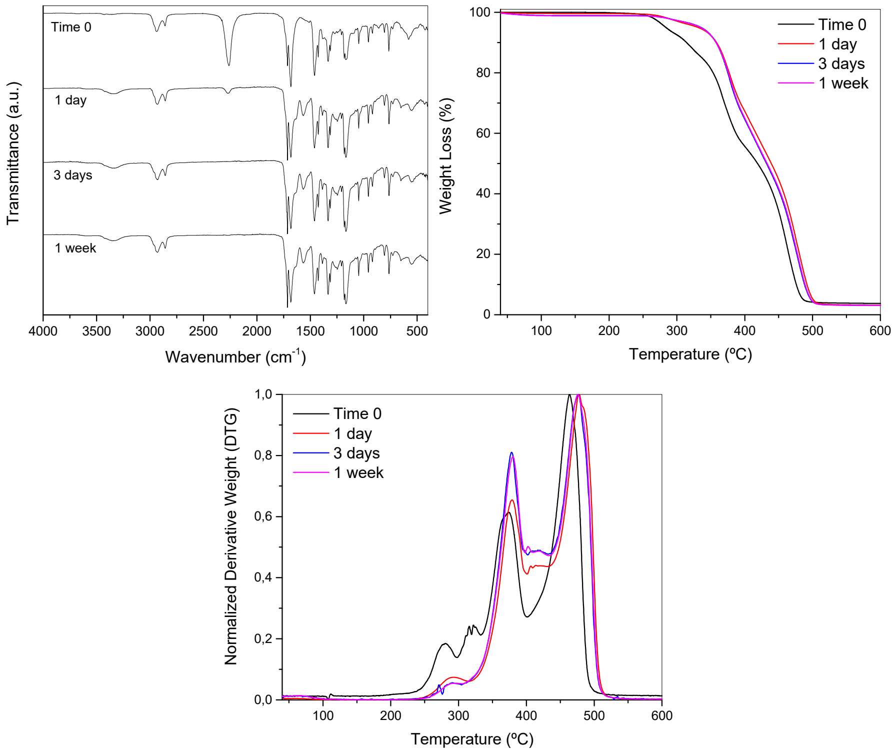

Figure S27. FTIR (top-left), TGA (top-right) and DTG (bottom) MCs-Polymer in water.

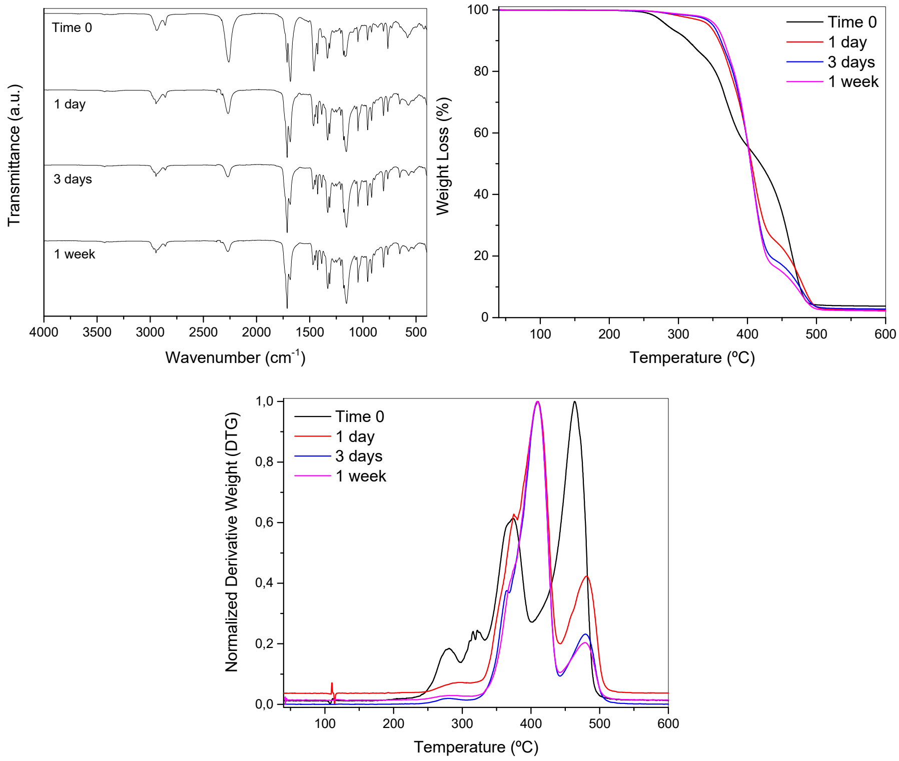

Figure S28. FTIR (top-left), TGA (top-right) and DTG (bottom) MCs-Polymer in acetone.

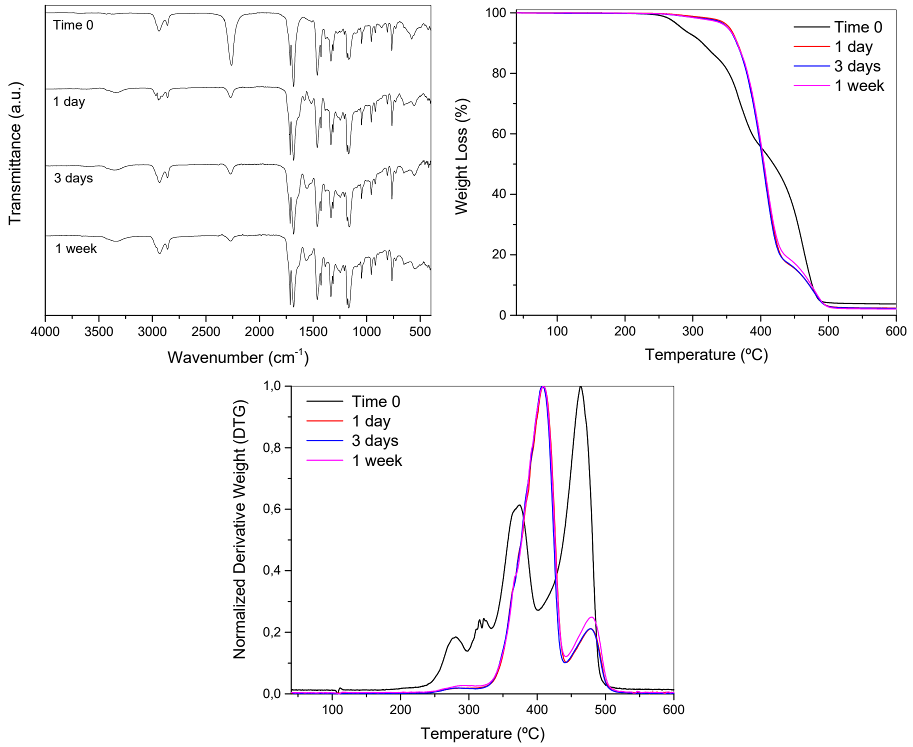

Figure S29. FTIR (top-left), TGA (top-right) and DTG (bottom) MCs-Polymer in ethyl acetate.

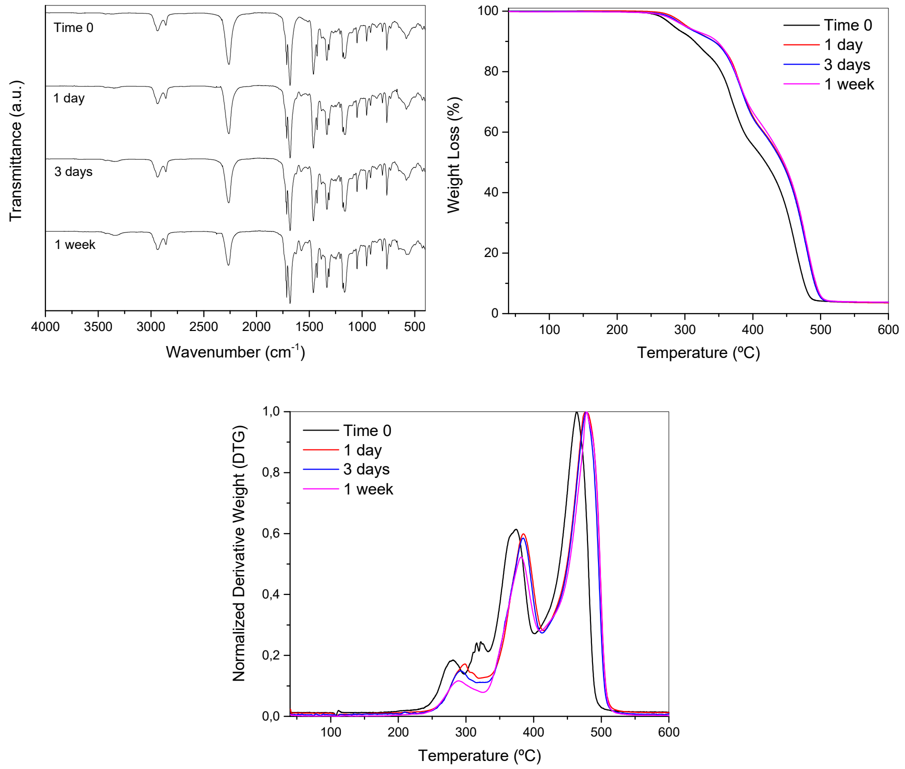

Figure S30. FTIR (top-left), TGA (top-right) and DTG (bottom) MCs-Polymer in hexane.

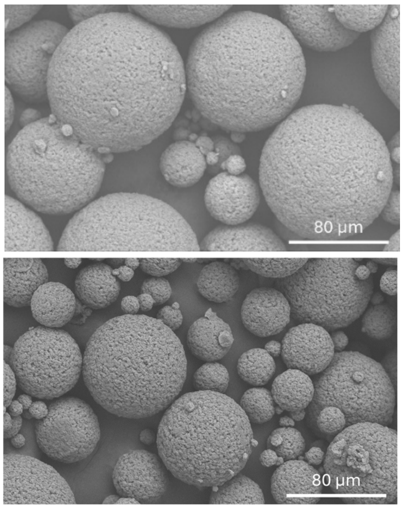

Figure S31. MCs with isocyanate after exposure to solvents after 1 week in water (top) and hexane (bottom)

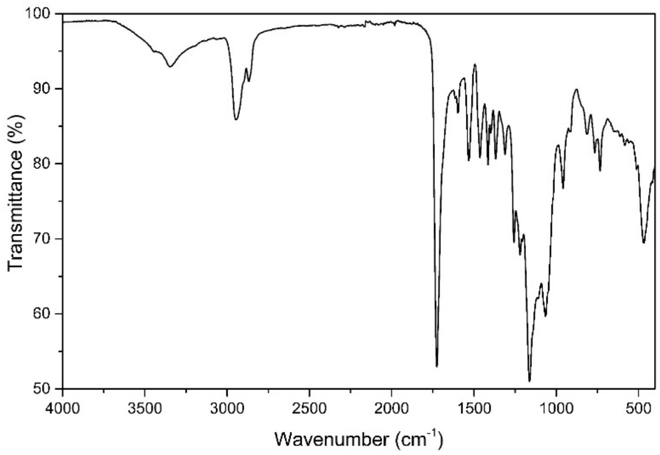

Figure S32. FTIR spectrum of the adhesive joint after 3 days of curing.

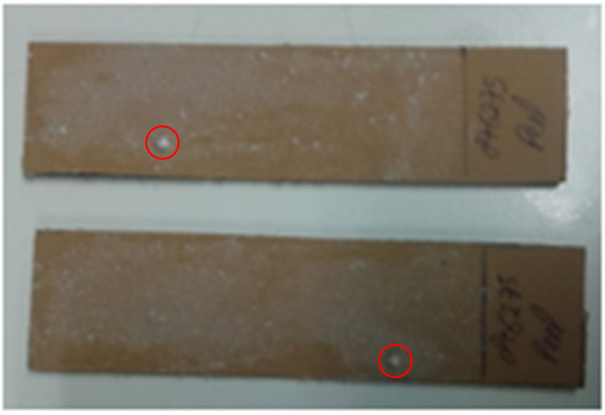

Figure S33. Lumps formation in the application of 6275 + MCs-Polymer in the substracts.

Table S4. Results from the peel strength tests with and without encapsulated isocyanates.

Sample	Average force/width (N/mm)	Maximum strength (N)	Average strength (N)	Average force/width (N/mm)
**Plastik 6275**	5.3	162.14	118.76	4.84 ± 0.43
	4.94	237.97	185.66	
	4.27	269.36	238.05	
**Monomer**	3.92	149.06	117.58	4.01 ± 0.09
	4.09	154.29	122.81	
**MCs-Monomer**	3.75	145.14	112.37	4.56 ± 0.61
	5.23	192.21	156.82	
	4.71	176.52	141.4	
**Trimer**	4.59	171.29	137.00	4.22 ± 0.28
	3.90	155.60	117.07	
	4.19	158.21	125.81	
**MCs-Trimer**	4.52	163.44	135.46	4.06 ± 0.45
	3.61	139.91	108.16	
**Polymer**	4.66	175.21	139.84	4.90 ± 0.37
	4.61	103.30	78.27	
	5.42	176.52	132.68	
**MCs-Polymer**	3.34	138.60	100.22	3.28 ± 0.19
	3.03	121.60	90.80	
	3.48	135.99	104.37	
